# COVID-19 and excess mortality in Russia: Regional estimates of life expectancy losses in 2020 and excess deaths in 2021

**DOI:** 10.1371/journal.pone.0275967

**Published:** 2022-11-02

**Authors:** Sergei Scherbov, Stuart Gietel-Basten, Dalkhat Ediev, Sergey Shulgin, Warren Sanderson

**Affiliations:** 1 Population and Just Societies Program, International Institute of Applied Systems and Analysis, Laxenburg, Austria; 2 Division of Social Science, The Hong Kong University of Science and Technology, Kowloon, Hong Kong SAR, China; 3 Department of Humanities and Social Science, Khalifa University of Science and Technology, Abu Dhabi, United Arab Emirates; 4 North-Caucasian State Academy, Institute for Applied Mathematics and Information Technologies, Cherkessk, Russia; 5 International Laboratory of Demography and Human Capital, Russian Presidential Academy of National Economy and Public Administration, Moscow, Russia; 6 Department of Economics, Stony Brook University, Stony Brook, NY, United States of America; University of Georgia, UNITED STATES

## Abstract

Accurately counting the human cost of the COVID-19 at both the national and regional level is a policy priority. The Russian Federation currently reports one of the higher COVID-19 mortality rates in the world; but estimates of mortality differ significantly. Using a statistical method accounting for changes in the population age structure, we present the first national and regional estimates of excess mortality for 2021; calculations of excess mortality by age, gender, and urban/rural status for 2020; and mean remaining years of life expectancy lost at the regional level. We estimate that there were 351,158 excess deaths in 2020 and 678,022 in 2021 in the Russian Federation; and, in 2020, around 2.0 years of life expectancy lost. While the Russian Federation exhibits very high levels of excess mortality compared to other countries, there is a wide degree of regional variation: in 2021, excess deaths expressed as a percentage of expected deaths at the regional level range from 27% to 52%. Life expectancy loss is generally greater for males; while excess mortality is greater in urban areas. For Russia as whole, an average person who died due to the pandemic in 2020 would have otherwise lived for a further 14 more years (and as high as 18 years in some regions), disproving the widely held view that excess mortality during the pandemic period was concentrated among those with few years of life remaining–especially for females. At a regional level, less densely populated, more remote regions, rural regions appear to have fared better regarding excess mortality and life expectancy loss–however, a part of this differential could be owing to measurement issues. The calculations demonstrate more clearly the true degree of the human cost of the pandemic in the Russian Federation.

## Introduction

Counting the human cost of the COVID-19 pandemic is clearly a demographic, policy and epidemiological priority. The particular circumstances in the Russian Federation have come to international attention because of the especially high levels of mortality compared to other parts of the world [1]. There are, however, many challenges to producing what appear to be a simple estimate of mortality derived from COVID-19. For example, differences in the adequacy and completeness of registration data around the world, as well as the definition and specification of causes of death represent a key challenge. According to the recommendations of the World Health Organization (WHO), death from COVID-19 is defined ‘for surveillance purposes as a death resulting from a clinically compatible illness, in a probable or confirmed COVID-19 case, unless there is a clear alternative cause of death that cannot be related to COVID disease (e.g. trauma)… There should be no period of complete recovery from COVID-19 between illness and death’ [2]. This definition, however, is not universally applied across all WHO member states, not least because of the challenges associated with determining a primary cause of death. The situation is even more complicated in the current pandemic, where a relatively mild infection can trigger a series of events resulting in death from an underlying condition. Such problems with defining cause of death result in wide variations in estimates of ‘deaths due to COVID-19’ at the global and national level [3].

In the Russian Federation (hereafter Russia), there appears to be an especially high degree of uncertainty concerning mortality due to COVID-19 [4]. The number of deaths due to COVID-19 reported by the Russian Federal State Statistical Service for 2020 (163,325) was three times that produced by the Russian Virus Response Center [5]. In other parts of the world, there have been marked differences between regions in terms of their mortality experience of COVID-19 [6,7]. Such uncertainty is expected to be amplified when considering the regional heterogeneity and different experiences of COVID-19 across the country [8].

Against this backdrop, the concept of ‘excess mortality’ has been set out as a possible ‘gold standard’ of measuring the human cost of the COVID-19 pandemic [9]. Excess mortality is estimated as the difference between the number of deaths that occurred during the COVID-19 pandemic in a particular period and the number of deaths that estimated to have occurred in the same period if there were no pandemic. The latter number is often referred to as the ‘baseline death count’. A major benefit of the excess mortality approach is that it is not sensitive to variations in cause of death reporting, and encompasses deaths relating directly to COVID-19 as well as those due to related causes and issues which may have stemmed from lockdowns, restriction on movement, postponed operations and so on [5]. This approach to measurement has been adopted in various datasets [10,11], by various international organizations (such as WHO [12], OECD [13], and by national statistical agencies (e.g. [14]).

Despite this consensus on the benefits of the excess mortality approach, there are still variations in the final calculated numbers of such deaths at the global and national level [3]. A recent paper by Kobak [8] produces various estimates for excess mortality stemming from COVID-19 in Russia, with one as high as 380,000 deaths (as of 1 January 2021), corresponding to 0.26% of the population. For the period April to November 2021, Kobak [8] estimates a total of 264,100 excess deaths (95% interval: [232,000, 296,200]), which is higher than the total number of COVID-19 related deaths reported to WHO for the entire period from January 2020 to December 2021 [15]. Kobak’s figures show Russia to have the second highest absolute number of estimated excess deaths in the world after the United States (over 400,000), and one of the highest numbers per capita. More recently, Aburto et al. [16] found that that life expectancy losses among females were the largest in Russia, and losses among males stand only behind the USA (2.2 years) and Lithuania (1.7 years), although Islam et al [20] estimate that Russian losses in life expectancy are highest for both men and women. Kobak [8] also produced estimates of excess mortality by Federal Region, and finds 23,500 cases of excess mortality in Moscow alone, with the highest excess mortality rate found in Samara Oblast: a 22% yearly mortality increase.

Part of the reason for the discrepancy in figures of excess mortality presented for Russia above stems from alternative methods of calculating this projected death count. As the recent study by Nepomuceno et al. [17] describes, there can be differences in measured outcomes based on a variety of factors such as the mortality index; the method used to estimate the baseline; the number of years included in the reference period; and the time unit of the death data (weekly and monthly). In addition, the jump-off effect of the previous year’s data for trend models must also be considered. Turning back to studies of excess mortality in Russia, in the first approach an average count of deaths for a particular time interval is calculated (e.g. five years before the pandemic began) and these numbers are compared to the number of deaths that occur during the pandemic [18]. In the second approach, used in Kobak [8], deaths are extrapolated. In this method, the average monthly number of deaths for each year are computed for 2006–19; then a linear trend is fitted to the 2015–2019 values which is then extrapolated to provide the predicted baseline value for 2020.

Neither approach accounts for changes in mortality and population age structure over time and can lead to a biased estimation of excess mortality from the COVID-19 pandemic. This can be especially true for an aging population with an irregular age structure–such as Russia. In each case, the dynamics of mortality and waves in the demographic structure will affect the assessment of deaths in the absence of a pandemic and, accordingly, affect the assessment of demographic losses due to the coronavirus pandemic.

For a more accurate assessment of the number of deaths in the absence of a pandemic, it is possible to use predicted values of the number of deaths that consider the peculiarities of the age structure of the population. To estimate the number of deaths in 2020–21 in the absence of a pandemic—and therefore to calculate excess mortality in comparison—it is necessary to make a population forecast for one year from the beginning of 2020 to 2021 or for two years from the beginning of 2020 to 2022. The population forecast can be made either with the mortality rate of 2019 or considering the dynamics of mortality in previous years to predict the expected mortality for 2020/2021. Both versions of the forecast allow us to estimate the number of deaths that would have occurred in 2020/2021 in the absence of the COVID-19 pandemic. Excess mortality, in this vein, is defined as the difference between the recorded number of deaths in 2020/2021 and the projected estimate of the number of deaths in the absence of the COVID-19. In this paper we followed the second approach. Projecting the number of deaths in the absence of the pandemic, we assumed that life expectancy would have increased at the same pace as it has been increasing recently. Such an approach was also performed in Islam et al. [19,20]. Timonin et al. calculated excess mortality for Russia and its regions for 2020 [5]. They found that Russia had one of the highest levels of excess mortality in the world and matched most closely those found in other central and eastern European countries. At the regional level, they found that excess mortality began to increase in Moscow and St. Petersburg in the Spring of 2020, and then spread to all regions of Russia by the end of 2020. They concluded by arguing that the observed negative association between excess mortality and reported cases was explained by under-recording of the latter.

In this paper, using the latest data released from ROSSTAT [19–22], we are able to calculate excess mortality for Russia and its regions for 2020 by age, sex, and rural-urban residence. For 2021, we produce estimates for regions only (because more granular data have not yet been released). In doing so, we build on the extant literature in the following ways: firstly, we are able to go beyond the estimates produced for 2020 in Kobak [8] by deploying this alternative methodology which takes into account projected changes in the age structure. Secondly, we produce the first estimates for 2021 using a variation of the enhanced method deployed by Timonin et al. [5] (building on Islam et al. [23]). Finally, we are able to go beyond the analysis performed in Timonin et al. [5] by producing estimates for rural and urban excess mortality for 2020.

Islam et al. [24] further applied an important measure of losses due to the pandemic, namely, the Years of Life Lost (*YLL*). Following WHO methodology [25], they calculated *YLL* based on expected remaining years of life from a standard life table for all countries. Such an approach, as useful as it may be for international comparisons, does not adequately express losses of expected years of life in countries like Russia where actual remaining life expectancies deviate substantially from those of the standard life table used in Islam et al. [23]. As such, we calculate a related indicator using life tables based on actual mortality data and extrapolations for Russia and its regions, relating the life years lost to those very people who perished due to the pandemic. To avoid confusion with *YLL*, we term our indicator Mean Remaining Life Expectancy of the Deceased (*RLED*).

## Materials and methods

The primary sources of data are derived from official ROSSTAT publications for the 2021 [19–22]; the Russian Economic School database [26] for the 2020; and calculations and population projections which were used in the Russian Demographic Data Sheet 2019 [27]. The database of the Russian Economic School provides death rates for 2020 by gender, region, urban and rural residence and single years of age as well as population exposures. The time series of observed life expectancies for the regions of Russia were obtained from Federal State Statistics Service [28].

Our approach builds on that of Islam et al. [20] who used the Lee-Carter model [29] to extrapolate age-specific mortality rates based on trends over the period 2005–2019. We do not, however, use that extrapolation method, because it relies on the existence of extended log-linear temporal trends of age-specific mortality rates, an assumption that may not be applied to the volatile and complex dynamics of mortality in Russia and its regions. Instead, we extrapolate the life expectancies at birth and then adjust the baseline 2019 death rates so as to fit to the extrapolated life expectancies (graduation and adjustment procedures come from an earlier projection for Russia [27]). Our method is somewhat similar to the one employed by UN [30,31] who also extrapolate life expectancy first and then disaggregate it into age-specific rates. There are important differences, though. In predicting the counterfactual life expectancy in the absence of the pandemic, given the short projection horizon and linear trends in the life expectancy since 2010, we use linear trend model fit to data for the years 2010 to 2019. To check relevance of the linear trend to our data, we calculated determination coefficients (R2). Among the regions included in our tabulations, the average determination coefficient was 94.1%, and only in one region (Arkhangelsk Region less autonomous area) was it below 70% (67%). When disaggregating the life expectancy into age-specific death rates, we use Ediev’s [32,33] approach to preserve consistent age-sex patterns of the projected mortality. To this end, we fit log-linear trends to age-sex-specific death rates in the period 2010–2019 (controlling for regional effects and interaction of age-sex with rurality)

$$\log\;M_{x,t}^{i}=a_{x,s}^{REG}+b_{i}+b_{x}t,$$

where $M_{x,t}^{i}$ – are the age-specific death rates in year *t* in population *i* (rates are calculated separately by region, rurality, and sex), $a_{x,s}^{REG}$ – age-sex- federal district (REG)-specific effects, *b*_*i*_−population-specific effects, *b*_*x*_−age-specific rates of mortality improvement over time. From the modelling outcomes, we obtained age-specific rates of mortality improvement similar to the *b*_*x*_ of the Lee-Carter model [25]. The age-specific rates of mortality improvement are then adjusted to form an age-monotone (increasing with age) profile $b_{x}^{*}$ that, being used in the projection, assures consistency of age profiles of the mortality rates. In the projection, the baseline death rates of 2019 are updated by applying the age-specific improvement rates $b_{x}^{*}$:

$$logM_{x,t}^{i}=logM_{x,t0}^{i}+k_{t}^{i}b_{x}^{*},$$

where $M_{x,t0}^{i}$ are the age-specific death rates in the base year; and $k_{t}^{i}$ are scaling factors that are matched to the projected life expectancy for a given population. The described procedure is technically similar to what has been recently introduced as the ‘rotation’ technique used, for example, in the UN’s *World Population Prospects* [34].

A standard cohort-component method-based system for producing multiregional population projections was used. To estimate excess mortality, we needed to project the deaths only for two years: 2020 and 2021. In this short time interval, the influence of migration and fertility on the total number of deaths would likely be insignificant (see below). Although we used a multiregional package to produce the number of deaths, similar results would have been obtained had we applied a cohort-component projection model to each region and urban settlement independently.

The population projection approach has several advantages over approaches that average the number of deaths over the previous five years or extrapolate the annual number of deaths from the years preceding pandemics. First, the change in the age structure of the population and its impact on the number of deaths is considered. Second, it is possible to reduce random fluctuations in the expected number of deaths, since the population forecasts produce graduated structures of mortality. In fact, the primary reason why the traditional method uses a five-year interval to average the number of deaths is the need to reduce the random component in estimating the number of deaths. Finally, because of the need to obtain time-series data on which to draw the projections—we used data from 2010- we excluded regions with short series of data available, and with small death count as described below (see Appendix for full list).

In this paper, to estimate excess mortality, we use a population forecast for all regions of Russia, that assumes the observed recent trend in life expectancy would have continued in the absence of the COVID-19 pandemic. The base population estimate is taken as of January 1, 2020. To estimate the number of excess deaths in 2020 and 2021, the population forecast is made for two years. As the impact of fertility and migration on the number of deaths in 2020–21 is extremely small, so the baseline scenario of fertility and migration, similar to the level of fertility and migration in 2019, was considered. Finally, to mitigate against high levels of fluctuations resulting from small numbers, we only consider populations where our calculated number of expected deaths is greater than 3,000 per year. Although this particular threshold was chosen rather arbitrarily, our decision was based on earlier experience with sampling errors of life expectancy estimates [34]. Given a Russian Crude Death Rate of roughly 12.8, 3,000 deaths correspond to around 235,000 people which, at life expectancy around 73 years, yields a sampling standard error of about 0.27 years (see Appendix 1 of [34]). This approximately corresponds to 95% CI (±0.5 years). Because the COVID-19 related losses to life expectancy are measured in years, we considered such a threshold to be reasonable for the current study.

To produce estimates of *RLED* in Russia and its regions in 2020 we use the following procedure. For each age group with non-negative excess mortality, we multiply the excess deaths by the corresponding remaining life expectancy in the absence of the COVID-19, sum up those products and divide by the total excess deaths in those age groups:

$$RLED=\frac{\sum_{x=0}^{100}(D_{x}^{obs}-D_{x}^{exp})*e_{x+.5}}{\sum_{x=0}^{100}\left(D_{x}^{obs}-D_{x}^{exp}\right)}$$

where $D_{x}^{obs}$ is the number of deaths that was observed in the age group *x* in 2020, $D_{x}^{exp}$ is the number of deaths that would be expected in the absence of COVID-19 in age group *x* in 2020, *e*_*x*_ is life expectancy at age *x* from the baseline life table in 2020 which would hold in the absence of pandemic. *RLED* is calculated for each region, urbanization status, and gender. We *do not estimate RLED* for 2021 because the required data have not been published yet.

Calculations were produced using packages written by authors in FORTRAN and R.

## Results

### Excess deaths at the regional level for 2020 and 2021

Based on our population projections, we estimate that there were 351,158 excess deaths in 2020 and 678,022 in 2021 in the Russian Federation (see Tables 1 and S1)—a total of 1,029,181 excess deaths over the period 2020–2021. This figure represents an excess of 19.65% and 38.36% over the expected number of deaths for 2020 and 2021 respectively. Our results are not much different from those that might have been obtained for Russia as a whole based on the official population projection by ROSSTAT that was conducted in 2019 [35]. Comparing deaths projected by ROSSTAT for 2020 and 2021 to actual figures, yield estimates of excess mortality 350,600 for 2020 and 657,800 for 2021 (1,007,400 in total over two years). ROSSTAT’s projection, however, do not provide the regional details that are studied here.

**Table 1:** Expected, observed and excess deaths (expressed in absolute and percentage terms), highest and lowest five regions of the Russian Federation with greater than 3,000 predicted deaths per year, 2021 and 2020, ranked by excess deaths as % of expected deaths.

Region	Expected deaths in thousands	Observed deaths	Excess deaths	Excess deaths as % of expected deaths
**2020**				
Chechen Republic	6.4	9.4	2.9	45.97
Republic of Dagestan	15.2	19.8	4.6	30.21
Republic of Tatarstan	42.3	54.3	12.0	28.41
Lipetzk oblast	15.8	20.2	4.4	27.92
Republic of Mordovia	10.3	13.1	2.8	26.95
*The Russian Federation*	*1*,*787*.*4*	*2*,*138*.*6*	*351*.*2*	*19*.*65*
Sakhalin oblast	5.9	6.7	0.8	13.07
Arkhangelsk Region (less NAA)	14.5	16.2	1.7	11.82
Zabaikalsk kray	12.9	14.4	1.5	11.58
Republic of Buryatia	10.6	11.8	1.2	11.26
Republic of Adygeya	5.6	6.2	0.6	10.37
**2021**				
Lipetzk oblast	15.5	23.6	8.1	52.14
Saratov oblast	32.4	49.1	16.6	51.25
Ryazan oblast	16.0	24.0	8.0	50.00
Orenburg oblast	24.7	36.6	11.9	48.00
Volgograd oblast	31.6	46.6	15.0	47.35
*The Russian Federation*	*1*,*767*.*5*	*2*,*445*.*5*	*678*.*0*	*38*.*36*
Moscow	132.5	172.8	40.3	30.39
Sakhalin oblast	5.8	7.6	1.8	30.29
Zabaikalsk kray	12.7	16.5	3.8	30.04
Kemerovo oblast	36.9	47.6	10.7	29.04
Primorsky kray	25.1	31.9	6.8	27.25

S1 Table shows the numbers of excess deaths per region for all regions with greater than 3,000 predicted deaths per year. Table 1 gives a snapshot of this regional data showing the five regions with the highest and lowest percentage of excess deaths. While the figures show a wide variation between provinces, no specific regional patterns (e.g. an east-west gradient) could be observed. In 2021, the highest percentages of excess mortality can be found in Lipetzk oblast (52.14%) in the Central Federal District, while the lowest can be found in Primorsky kray (27.25%) in the Far East of the country. In terms of the regional distribution, the patterns are far from clear cut. In 2020, for example, the range was from an 10.37% excess in the Republic of Adygeya in the Northern Caucasus up to a 45.97% excess in the Chechen Republic. In other words, some of the highest and lowest percentages of excess mortality can be found in the Caucasus–a feature which warrants further investigation. Similarly, while four of the six regions with the lowest excess mortality (Republic of Buryatia, Sakhalin oblast, Zabaikalsk kray and Promorsky oblast) can be found in the Far Eastern Federal District, so too can the 9^th^ *highest* ranking region (Republic of Sakha). Indeed, the regions with the highest excess mortality in 2021 are to be found around the country: four in the Central Federal District (Lipetzk oblast, Ryazan oblast, Voronezh oblast and Kursk oblast); three in the Volga Federal District (Saratov oblast, Orenburg oblast and Republic of Mordovia) and one each in the Southern Federal District (Volgorad oblast), Northwestern Federal District (Republic of Karelia) and Far Eastern Federal District (Republic of Sakha). Among the regions with the lowest excess mortality, the majority are to be found in the sparsely populated regions of the Far Eastern Federal District and Siberia. Moscow City, however, is also in this low ranked groups.

Finally, there is a high degree of change both between the two years and across the regions of Russia. In the Republic of Karelia, for example, excess mortality rose from 17.11% in 2020 to 46.3%, propelling it from a middle-ranking position to one of the highest in the country. A further five regions saw increases in excess mortality of greater than 25% (Kursk oblast, Kostroma oblast, Novgorod oblast, Voronezh oblast and Saratov oblast). Other states, meanwhile, saw much less dramatic changes. Eleven regions saw increases of 10–15% (Khabarovsk kray, Kabardian-Balkar Republic, Primorsky kray, Novosibirsk oblast, Chelyabinsk oblast, Chuvash Republic, Samara oblast, Udmurt Republic, Perm kray, Republic of Bashkortostan, and Kemerovo oblast). Excess mortality in the Republic of Dagestan, meanwhile, stayed almost constant between 2020 and 2021. Because of the sharp increases across the country, this meant the region dropped in the national rankings from second top to eighth last. Mortality in the Chechen Republic appears to have *fallen* between 2020 and 2021.

### Excess deaths and life expectancy loss at the regional level for 2020 by urban-rural residence, gender and age

S2 Table shows excess mortality in urban and rural areas. As expected, the percentage of excess deaths is higher in urban areas due to the much higher population density. A summary of this table is presented in Table 2. In 2020, there were 274,520 excess deaths in urban areas compared to 76,640 in rural areas. Our results for Russia are consistent with other studies [16,20]. The Republic of Dagestan has the highest excess mortality of either rural or urban areas (34.8%), and the highest number of years in terms of life expectancy lost (3.5). Despite this, it is apparent that the highest levels of excess mortality were, indeed, higher in the urban areas than in rural areas. The top five urban areas (Lipetzk oblast, Republic of Mordovia, Churvash Republic, Republic of Tatartstan and Orenburg oblast) all reported excess mortality greater than 30% than expected mortality. In rural areas, meanwhile, only the Republic of Dagestan reported excess mortality of greater than 30%; the remaining four areas in the top five (Samara Oblast, Kabardian-Blakar Republic, Astrakhan oblast and Leningrad oblast) all reported excess mortality between 23.7 and 24.7. The lowest excess mortality in urban areas was found in Vologda oblast, Republic of Buryatia, Sakhalin oblast, Moscow city and Kaliningrad oblast. In these areas, excess mortality was between 13.6% and 15.3%. In the ‘best performing’ rural areas, however (Irkutsk oblast, Kemerovo oblast, Zabaikalsk oblast, Arkhangelsk Region less autonomous area and the Chechen Republic), excess mortality was comparatively much lower (below 6%). Rural areas within the Chechen Republic, in fact, reported *negative* excess mortality. However, these data bring out one of the main caveats relating to measurements of excess mortality in rural areas–namely that deaths may have been registered in urban areas. This, then, will lead to an inflation of urban excess mortality, and an undercount of rural mortality. As such, extreme caution should be exercised in the interpretation of these results.

**Table 2:** Expected, observed and excess deaths (expressed in absolute and percentage terms), highest and lowest five regions of the Russian Federation with greater than 3,000 predicted deaths per year, 2020, urban and rural areas.

Region	Excess deaths in thousands	Excess deaths as a percent of expected	Life expectancy loss
**URBAN**			
Lipetzk oblast	3.04	32.6	2.9
Republic of Mordovia	1.73	31.1	2.4
Chuvash Republic	2.35	31.0	2.3
Republic of Tatarstan	9.06	30.8	2.5
Orenburg oblast	4.30	29.5	2.4
*The Russian Federation*	*274*.*52*	*21*.*2*	*1*.*9*
Vologda oblast	1.60	15.3	1.0
Republic of Buryatia	0.86	15.2	1.0
Sakhalin oblast	0.67	14.5	1.0
Moscow city	17.72	13.7	0.9
Kaliningrad oblast	1.27	13.6	0.6
**RURAL**			
Republic of Dagestan	3.19	34.8	3.5
Samara oblast	2.15	24.7	2.9
Kabardian-Balkar Republic	0.81	24.5	2.6
Astrakhan oblast	0.84	23.8	2.4
Leningrad oblast	1.51	23.7	3.6
*The Russian Federation*	*76*.*64*	*15*.*5*	*2*.*0*
Irkutsk oblast	0.39	5.6	1.3
Kemerovo oblast	0.29	5.3	1.4
Zabaikalsk kray	0.19	4.0	1.1
Arkhangelsk Region less autonomous area	0.02	0.4	0.6
Chechen Republic*	-0.01	-0.4	-0.1

* Note: For the case of the Chechen Republic (as also in some other areas), it is likely that COVID-19 deaths were registered in urban areas, hence leading to very low levels of excess mortality. However, the urban data is not described here as the expected number of deaths in urban area was less then 3000.

S3 Table show the excess deaths and life expectancy lost by gender for the regions of Russia. This is summarized in Table 3, which again gives a snapshot of variation showing the regions with the highest and lowest mortality by gender. Nationally in 2020, there were 171,600 excess deaths for males compared to 179,560 for females. For both males and females, the Chechen Republic returns the highest percentage of excess mortality (48.2% for males, 43.5% for females). For males, the next highest percentages of excess mortality can be found in Republic of Dagestan (33.1%), Lipetzk oblast (28.0%), Republic of Tatarstan (27.7%), and Moscow oblast (25.7%); while the lowest can be found in Kaliningrad oblast (12.3%), Sakhalin oblast (12.1%), Moscow city (10.1%), Republic of Buryatia (10.1%) and Zabaikalsk kray (9.9%). For females, the highest excess mortality (after Chechen Republic) can be found in Khanty-Mansi Autonomous Area–Yugra (32.9%), Orenburg oblast (30.2%), Republic of Tatarstan (29.1%), and Samara oblast (28.4%). The lowest, meanwhile, can be found in Republic of Komi (13.1%), Ivanovo oblast (12.8%), Republic of Buryatia (12.6%), Novgorod oblast (11.5%), and Arkhangelsk Region less autonomous area (10.8%). The data show that while the differences by gender at the national level were modest (comparing 20.1% with 19.2% excess mortality for males and females respectively), this masks important regional differences. For example, in Moscow City, the percentage of excess deaths for males is one of the very lowest in the country at 10.1%. For females, however, it is closer to the national average at 17.7%. In the Khanty-Mansi Autonomous Area, meanwhile, the gap is even greater, 21.6%, for females and 32.9% for males.

**Table 3:** Expected, observed and excess deaths (expressed in absolute and percentage terms), highest and lowest five regions of the Russian Federation with greater than 3,000 predicted deaths per year, 2020, males and females.

Region	Excess deaths in thousands	Excess deaths as a percent of expected	Life expectancy loss
**MALES** Chechen Republic	1.63	48.2	4.7
Republic of Dagestan	2.63	33.1	3.9
Lipetzk oblast	2.17	28.0	3.3
Republic of Tatarstan	5.93	27.7	3.0
Moscow oblast	11.07	25.7	2.9
*The Russian Federation*	*171*.*60*	*19*.*2*	*2*.*2*
Kaliningrad oblast	0.70	12.3	0.9
Sakhalin oblast	0.39	12.1	1.0
Moscow city	6.53	10.1	0.7
Republic of Buryatia	0.58	10.1	0.7
Zabaikalsk kray	0.71	9.9	0.9
**FEMALES**			
Chechen Republic	1.32	43.5	3.8
Khanty-Mansi Autonomous Area–Yugra	1.36	32.9	2.7
Orenburg oblast	3.69	30.2	2.9
Republic of Tatarstan	6.08	29.1	2.5
Samara oblast	5.96	28.4	2.7
*The Russian Federation*	*179*.*56*	*20*.*1*	*2*.*0*
Republic of Komi	0.60	13.1	1.0
Ivanovo oblast	1.06	12.8	1.1
Republic of Buryatia	0.62	12.6	1.4
Novgorod oblast	0.57	11.5	1.2
Arkhangelsk Region less autonomous area	0.76	10.8	0.7

**Fig 1:**
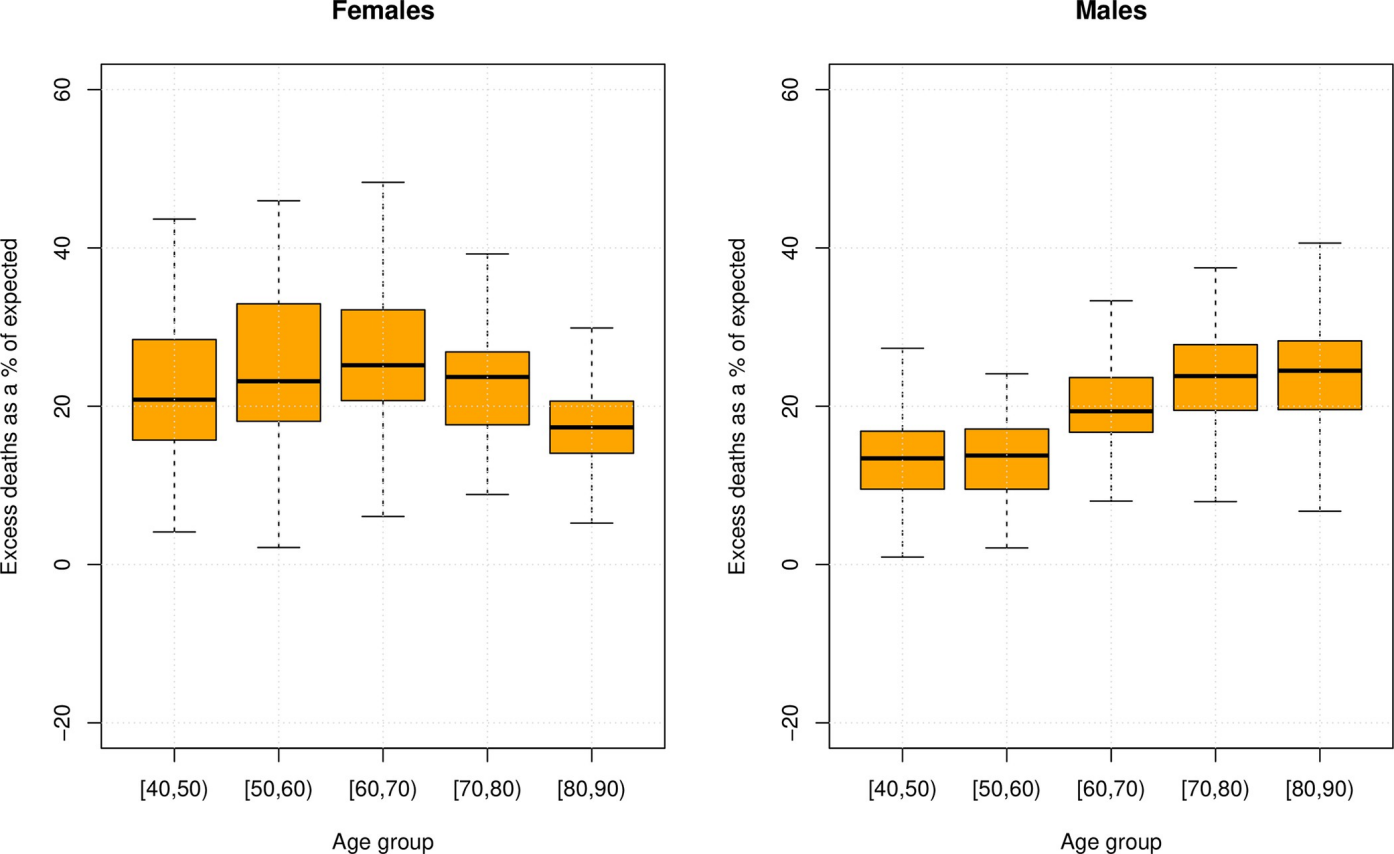
Boxplot of excess mortality of regions (unweighted), males and females, ages 40–90, Russian Federation 2020.

Next, we turn to the distribution of excess mortality by age. This is represented in Fig 1 –a box plot of excess deaths as a percentage of the expected number of deaths, with the unit of measurement distribution being the regional territories of Russia. Fig 1 shows a number of distinct patterns. Firstly, we see a different relationship between age and excess mortality for males and females. For males, the relationship is generally linear. For females, however, there is an increase from age group 40–50 to 60–70, but then a dropping away towards the highest ages. Turning to the distribution, we can see there is a high degree of variation between regions for both males and females. However, it is clear that the range of the distribution is higher for females than for males. S4 Table shows excess mortality for those aged above and below 65. The details for other age groups are available on request from the authors.

### Mean Remaining Life Expectancy of Deceased (RLED)

Finally, we present the results for the Mean Remaining Life Expectancy of the Deceased *(RLED)* for all regions in S5 Table. Table 4 shows the top and bottom five regions by *RLED* for 2020. For males in two regions (Khanty-Mansi Autonomous Area–Yugra and Kabardian-Balkar Republic), this *RLED* was greater than 17 years, and in the Republic of Sakha in the Far East it was 18.4. For females, meanwhile, three regions (Republic of Buryatia and Khanty-Mansi Autonomous Area–Yugra) saw *RLED* greater than 16 years while, again, the Republic of Sakha had the highest *RLED* at 17.9 years. Other regions, meanwhile, saw lower levels of *RLED*. Amongst males, Khabarovsk kray reported a *RLED* of 12.4 years, while Omsk oblast, Chelyabinsk oblast, Republic of Buryatia and Altai kray all reported *RLED*s between 10.9 and 11.8 years. For females, the lower *RLED* in Omsk oblast, Republic of Tatarstan, Udmurt Republic, Chelyabinsk oblast and Kirov oblast were all rather narrowly distributed between 11.9 and 12.2 years.

**Table 4:** Remaining life expectancy of the deceased, RLED, in regions of the Russian Federation with greater than 3,000 predicted deaths per year, 2020, males and females.

Region	Excess deaths as a percent of expected	*RLED*
**MALES**		
Republic of Sakha (Yakutia)	22.5	18.4
Khanty-Mansi Autonomous Area–Yugra	22.6	17.2
Kabardian-Balkar Republic	25.8	17.1
Republic of Dagestan	34.4	16.8
Leningrad oblast	20.9	16.5
*The Russian Federation*	*19*.*5*	*13*.*8*
Khabarovsk kray	19.2	12.4
Omsk oblast	24.1	11.8
Chelyabinsk oblast	22.5	11.8
Republic of Buryatia	10.7	11.7
Altai kray	18.0	10.9
**FEMALES**		
Republic of Sakha (Yakutia)	21.8	17.9
Republic of Buryatia	12.7	16.3
Khanty-Mansi Autonomous Area–Yugra	33.9	16.3
Republic of Dagestan	28.2	16.0
Kabardian-Balkar Republic	20.1	15.3
*The Russian Federation*	*20*.*4*	*12*.*9*
Omsk oblast	25.7	12.2
Republic of Tatarstan	29.6	12.1
Udmurt Republic	21.4	12.1
Chelyabinsk oblast	23.8	12.0
Kirov oblast	20.8	11.9

Table 4 shows clearly how the data dramatically diverge from the widespread notion that the pandemic might have had a limited effect on people’s lifespans, because it primarily kills those who would anyway die in a near future. For Russia as whole, an average person who died due to the pandemic in 2020, would have otherwise lived for almost a further 14 more years. Our RLED estimates are relatively similar to estimates for other countries. Based on evidence from the Human Mortality Database [36], we estimate RLED for Sweden as 8.1 years in 2020 and 16.2 years in 2021.

## Discussion

Our calculations found significant degrees of regional divergence in terms of the experience of COVID-19 related excess mortality. This is important for two reasons. Firstly, this reveals the very unequal experience of COVID-19 across the country and may reflect the heterogeneity of the degree to which medical and public health systems at the regional level may have been affected by the pandemic and other local factors which shaped the regional pandemic experience [37,38]. Secondly, this regional divergence further justifies our methodological approach. Given the fact that the regions of Russia differ substantially in age structure and the dynamics and level of life expectancy, the use of the average number of deaths, as an estimate of the number of deaths in the absence of a pandemic, can lead to a distortion in the estimation of excess mortality. Naïve linear extrapolations of deaths, similarly, may be biased in regions, like Moscow, where the combination of population aging and mortality decline results in complex non-monotonic changes in the number of deaths. Indeed, due to the age structure shifts of the population of Russia, it is expected that the number of deaths will start rising soon, also making linear trend assumptions questionable. While historic trends were demonstrating a close to linear decline in the number of deaths on the national level, there is no evidence that this is happening on the regional level. Furthermore, the estimates of expected deaths using linear regression (and any other type of regression as well) depend on the length of historical time series used in the regression and is affected by outliers in the observations where, in the case of Russia, years like 2010 and 2017 returned relatively low numbers of deaths).

Our results point to considerable regional variation in excess mortality that deserves further analysis. Tentatively, one may note several factors that might have played their role in forming the regional differences in losses to the pandemics. First, we find that there is substantial positive correlation between the regional demographic losses and the life expectancy projected in the absence of the pandemic. Both life expectancy loss and excess mortality show correlation with the projected life expectancy of around 0.45 for males and 0.57 for females (Table 5). On this basis, one may speculate that regions leading in life expectancy improvements before the pandemic may have suffered more because of accumulation of population with poorer health status who were hit harder during the pandemic. The pandemic appears to have played a levelling role by cutting down the advantages in life expectancy of regions leading in mortality reduction.

**Table 5:** Correlation (Pearson *r* with p-values) of expected life expectancy with 2 indicators, all regions of Russia, 2020.

Indicator	Female	Male
Excess deaths as a percent of expected	0.44 (1.2e-4)	0.58 (7.6e-8)
Loss in life expectancy	0.46 (4.4e-5)	0.57 (1.5e-7)

Note: Numbers in the parentheses indicate p-values.

Secondly, in addition to purely demographic factors, one may also expect socio-cultural, economic and, perhaps, geographic differentials also to contribute to differences in the impact of the pandemics. Regions of the Northern Caucasus, for example, are known for their tradition of elderly living in larger households of extended families together with their children and descendants. While this is generally considered a health-protective behavior, such a tradition might have contributed to higher social exposure and, hence, higher losses in the Northern Caucasus.

A preliminary analysis of association between average regional income in 2019 was performed, and number of beds per capita in regional hospitals in 2019 as independent variables and regional excess deaths as a percent of expected as dependent. We run a multiple regression for 2020, 2021 and a combination of 2020–21 data. In all cases the influence of regional income was statistically insignificant, while number of hospital beds per capita was significant at 0.001 level in 2020 while nonsignificant in 2021. An explanation could be that in 2021, the number of hospital beds drastically increased in the regions due to temporarily organized treatment facilities and thus linking excess death in 2021 with the number of hospital beds per capita loses its meaning. Correlation between the number of hospital beds per capita and excess mortality in 2020 was -0.37 and significant at p-value = 0.001 level. We also found a negative correlation between female education and excess mortality in 2020, but no such correlation with the male education, although these results are preliminary and need further investigation. We intend a thorough study of factors of regional variation in excess mortality in the future.

Of course, infrastructure of the region—especially in terms of health facilities—would also contribute to the differences in excess mortality. There is, for instance, a huge disparity in excess mortality between Moscow city and the surrounding Moscow oblast. Life expectancy in Moscow oblast is considerably lower than in Moscow (expected life expectancy for males 67.7 versus 73.1). Moscow city, following the arguments introduced above, should have higher excess mortality. On the contrary, excess deaths measured as a percent of expected for men in 2020 was more than two-time higher in Moscow oblast compared to Moscow (25.7% versus 10.1%). Residents of these two places are not especially different in terms of culture, although that does not mean that there are not large behavioral differences. Health care in Moscow city is more advanced than in Moscow oblast, but under normal circumstances for advanced medical treatments people in Moscow oblast would not need to travel far to get treatment in Moscow city. However, in the case of a pandemic, when most health facilities in Moscow were running at (or near) full capacity, there were fewer opportunities for patients from Moscow oblast to be treated in Moscow city.

Policies to prevent the spread of COVID-19 and the implementation of measures also played a role in the number of infections. The central government delegated much of the responsibilities in this field to regional administrations and those policies were diverse in the regions of Russia. At the initial stage of the COVID-19 pandemic, there was a high degree of misinformation related to the spread and severity of COVID-19 in Russian media. Sometimes public figures and even medical doctors on TV programs downplayed the danger of COVID-19 [39]. That influenced the efficiency of vaccination policies which, in turn, contributed to the number of COVID-19 cases and the excess number of deaths. A recent study by Roschina et al. has highlighted a high degree of ‘vaccine hesitancy’ in Russia, with only 45% of those surveyed in 2021 demonstrating positive attitudes towards the vaccination program [40]. As of March 2022, the total vaccination rate remains relatively low in Russia (around 55%, with around 20% in some regions) [41]. This compares unfavourably to even the global figure of around 60%, and is lower than in Pakistan and The Philippines. Maleva et al. [42] found that Russian young people and people with low levels of education are the least likely to be vaccinated.

So, in general there are many social, political, cultural and epidemiological factors and their interactions contributed to the diversity of patterns of excess mortality. In each region, the combination of those factors must be studied. In the Chechen Republic, for example, we observed a sharp drop in excess mortality between 2020 and 2021. This may be related to the hardline rhetoric and measures put in place in 2021 by the Chechen leader Ramzan Kadyrov. In May 2021, for example, it is reported that Kadyrov warned citizens that those who refused to be vaccinated would be treated last and will ‘feel how terrible the lack of oxygen feels’ [43]. Meanwhile in July 2021, it was decreed that unvaccinated residents in the Chechen Republic were banned from attending mosques. Citizens also stated that it was impossible to buy bread without a vaccine certificate [44].

Stepping back, our data presents clear evidence of the very high levels of excess mortality seen in Russia over the past two years; and that this level has significantly accelerated between 2020 and 2021. In a comparative sense, this overall finding accords with previous analyses of Russia which suggest it may come out of the COVID-19 experience with one of the very highest levels of excess mortality in the world. Using our *RLED* measure, however, we clearly disprove the widely held concept that such deaths were ‘going to happen anyway’ because of the relatively large number of remaining years of life expectancy of a deceased. Together, it appears that COVID-19 has further added to traumatic recent history of mortality–especially among males–in Russia [45,46].

Our study does, however, have several limitations. Firstly, the restricted data we have for 2021 means we cannot provide an analysis on the level of granularity which we would wish for this year. Secondly, as with all studies of mortality in Russia, there will inevitably be challenges concerning both completeness of registration as well as discrepancies in the place of registration of a death because of migration and different living/working patterns [47]. This is especially likely to be the case of the Republics in the Caucasus, and perhaps also the areas surrounding major cities such as Moscow and St. Petersburg. Such issues relating to death registration also impact upon the potential validity of comparisons between urban and rural excess mortality (as discussed above).

## Conclusion

The human cost of the COVID-19 pandemic is well known. Quantifying this carefully, however, is a high priority to not only measure this impact, but to ensure adequate policy responses. In this paper, we have identified the high levels of excess mortality in the regions of Russia during the COVID-19 pandemic. To do so, we have deployed a rigorous method which accounts more accurately for underlying demographic change. We estimate that more than a million lives were lost to the pandemic. Contrary to the popular belief that such deaths were ‘likely to occur anyway’, our analysis shows that COVID-19 in Russia has had a significant impact upon remaining life expectancy. We have also, for the first, time, been able to present regional figures of excess mortality for 2021, as well as an analysis by age, gender and urban and rural status. These figures showed a higher impact on life expectancy for males who also had rising excess mortality (as a percent of expected) with age, unlike females where there was a non-linear relationship, and in general higher level of excess mortality in urban areas compared to rural areas and little difference is observed in male versus female excess mortality. Higher urban excess mortality could be driven by a higher density of population in urban areas, and thus higher infection transmission rate. As mentioned earlier, another source of bias towards higher urban excess mortality may be caused by registration of deaths of rural residents transferred to urban hospitals. While there is a wide variation in mortality rates between regions, there is no clear regional distribution (e.g. an east-west gradient).

While national figures show that excess mortality in Russia is perhaps amongst the highest in the world, there is a wide degree of regional variation. Such variation–and its interaction with age, gender, and rural/urban status–is key to better consideration and formulation of public health strategies to mitigate both the ongoing impact of COVID-19, and to rebuild and reshape health systems after the pandemic is over. As such, the determinants of mortality and the experience of (and response) to the pandemic at the regional level is a critical future avenue of research. This would enable more precise policy recommendations to be generated from our research. At this stage, however, some outcomes are clear. The regional disparities in infrastructure–especially in health–appear to be a key factor in determining levels of excess mortality. These disparities become amplified when the circumstances of the pandemic disrupted access. Higher levels of excess mortality in urban areas may have resulted from greater levels of infection in spaces of higher population density. This may mean that alternative strategies for infection control and management need to be in pace for urban and rural areas. Finally, we must look at the broader picture of how such mortality is so high in the first place. In this, the role of (mis)information and communication concerning both the severity of the public health emergency appears to have played a critical role. Ensuring the public have access to objective, honest, impartial, and accurate information about their circumstances is of the highest priority. For their part, scientists should be providing clear transparent assessments of mortality to inform government, media and other stakeholders.

## Supporting information

S1 Table Expected, observed and excess deaths (expressed in absolute and percentage terms), highest and lowest five regions of the Russian Federation with greater than 3,000 predicted deaths per year, 2021 and 2020. (DOCX)

S2 Table Expected, observed and excess deaths (expressed in absolute and percentage terms) and life expectancy lost, regions of the Russian Federation with greater than 3,000 predicted deaths per year, 2020, urban and rural areas. (DOCX)

S3 Table Expected, observed and excess deaths (expressed in absolute and percentage terms), regions of the Russian Federation with greater than 3,000 predicted deaths per year, 2020, males and females. (DOCX)

S4 Table Expected, observed and excess deaths (expressed in absolute and percentage terms), regions of the Russian Federation with greater than 3,000 predicted deaths per year, 2020, aged below and above 65. (DOCX)

S5 Table Remaining life expectancy in regions of the Russian Federation with greater than 3,000 predicted deaths per year, 2020, males and females. (DOCX)
