# Supplementary material for: COVID-19 and excess mortality in Russia: Regional estimates of life expectancy losses in 2020 and excess deaths in 2021
Source: PLoS One. 2022 Nov 2;17(11):e0275967. doi: 10.1371/journal.pone.0275967 (PMC9629588; doi:10.1371/journal.pone.0275967)
Supplement: S1 Table — (DOCX) [file pone.0275967.s001.docx]

**S1 Table: Expected, observed and excess deaths (expressed in absolute and percentage terms), highest and lowest five regions of the Russian Federation with greater than 3,000 predicted deaths per year, 2021 and 2020.**

1. 2021

| ROSSTAT Code | Region | Expected deaths | Observed deaths | Excess deaths | Excess death as percentage of expected in % |
| --- | --- | --- | --- | --- | --- |
| 1142 | Lipetzk oblast | 15.5 | 23.6 | 8.1 | 52.14 |
| 1163 | Saratov oblast | 32.4 | 49.1 | 16.6 | 51.25 |
| 1161 | Ryazan oblast | 16.0 | 24.0 | 8.0 | 50.00 |
| 1153 | Orenburg oblast | 24.7 | 36.6 | 11.9 | 48.00 |
| 1118 | Volgograd oblast | 31.6 | 46.6 | 15.0 | 47.35 |
| 1120 | Voronezh oblast | 31.8 | 46.5 | 14.7 | 46.37 |
| 1186 | Republic of Karelia | 8.5 | 12.5 | 3.9 | 46.28 |
| 1138 | Kursk oblast | 16.1 | 23.3 | 7.2 | 44.74 |
| 1198 | Republic of Sakha (Yakutia) | 7.3 | 10.6 | 3.3 | 44.46 |
| 1189 | Republic of Mordovia | 10.1 | 14.6 | 4.5 | 44.30 |
| 1192 | Republic of Tatarstan | 41.8 | 60.3 | 18.5 | 44.23 |
| 1188 | Republic of Mariy El | 7.8 | 11.2 | 3.4 | 44.01 |
| 1156 | Penza oblast | 17.9 | 25.6 | 7.7 | 43.09 |
| 1134 | Kostroma oblast | 8.8 | 12.6 | 3.8 | 43.06 |
| 1152 | Omsk oblast | 23.8 | 33.8 | 10.0 | 42.12 |
| 1178 | Yaroslavl oblast | 17.7 | 25.1 | 7.4 | 42.10 |
| 1112 | Astrakhan oblast | 11.4 | 16.2 | 4.8 | 42.09 |
| 1173 | Ulyanovsk oblast | 16.7 | 23.8 | 7.0 | 42.04 |
| 1115 | Bryansk oblast | 16.8 | 23.8 | 7.0 | 41.67 |
| 1146 | Moscow oblast | 88.7 | 125.7 | 36.9 | 41.64 |
| 1122 | Nizhny Novgorod oblast | 44.4 | 62.9 | 18.5 | 41.63 |
| 1168 | Tambov oblast | 14.6 | 20.7 | 6.1 | 41.62 |
| 1117 | Vladimir oblast | 20.3 | 28.6 | 8.4 | 41.37 |
| 1147 | Murmansk oblast | 8.2 | 11.5 | 3.4 | 41.08 |
| 1171100 | Khanty-Mansi АА – Yugra | 10.2 | 14.4 | 4.2 | 41.03 |
| 1136 | Samara oblast | 41.1 | 57.8 | 16.7 | 40.54 |
| 1166 | Smolensk oblast | 13.4 | 18.8 | 5.4 | 40.52 |
| 1197 | Chuvash Republic | 14.6 | 20.5 | 5.9 | 40.49 |
| 1149 | Novgorod oblast | 9.4 | 13.1 | 3.8 | 40.22 |
| 1141 | Leningrad oblast | 23.0 | 32.2 | 9.2 | 40.06 |
| 1154 | Oryol oblast | 10.8 | 15.1 | 4.3 | 39.88 |
| 1133 | Kirov oblast | 17.4 | 24.3 | 6.9 | 39.79 |
| 1103 | Krasnodar kray | 69.8 | 97.1 | 27.3 | 39.14 |
| 1169 | Tomsk oblast | 11.9 | 16.5 | 4.6 | 39.02 |
| 1196 | Chechen Republic | 6.4 | 8.9 | 2.5 | 38.87 |
| 1180 | Republic of Bashkortostan | 48.0 | 66.4 | 18.4 | 38.31 |
| 1140 | Saint Petersburg | 61.0 | 84.2 | 23.2 | 38.05 |
| 1129 | Kaluga oblast | 14.1 | 19.5 | 5.4 | 37.99 |
| 1127 | Kaliningrad oblast | 11.6 | 16.0 | 4.4 | 37.49 |
| 1170 | Tula oblast | 22.3 | 30.7 | 8.3 | 37.31 |
| 1101 | Altai kray | 31.9 | 43.7 | 11.8 | 36.93 |
| 1125 | Irkutsk oblast | 30.5 | 41.7 | 11.2 | 36.89 |
| 1187 | Republic of Komi | 9.5 | 13.1 | 3.5 | 36.81 |
| 1190 | Republic of North Ossetia - Alania | 7.2 | 9.8 | 2.6 | 36.69 |
| 1128 | Tver oblast | 19.5 | 26.6 | 7.1 | 36.61 |
| 1158 | Pskov oblast | 9.9 | 13.5 | 3.6 | 36.30 |
| 1175 | Chelyabinsk oblast | 44.4 | 60.6 | 16.1 | 36.23 |
| 1137 | Kurgan oblast | 12.3 | 16.6 | 4.4 | 35.67 |
| 1110 | Amur oblast | 10.5 | 14.3 | 3.8 | 35.62 |
| 1165 | Sverdlovsk oblast | 56.7 | 76.9 | 20.2 | 35.60 |
| 1107 | Stavropol kray | 31.2 | 42.3 | 11.0 | 35.35 |
| 1191 | Republic of Karachaevo-Chercassia | 4.2 | 5.7 | 1.5 | 35.11 |
| 1119 | Vologda oblast | 15.6 | 21.1 | 5.5 | 35.11 |
| 1104 | Krasnoyarsk kray | 34.9 | 47.1 | 12.2 | 35.04 |
| 1111001 | Arkhangelsk Region (less NAA) | 14.3 | 19.3 | 5.0 | 34.83 |
| 1194 | Udmurt Republic | 17.3 | 23.3 | 6.0 | 34.41 |
| 1171001 | Tyumen Region (less AAs) | 16.1 | 21.5 | 5.4 | 33.65 |
| 1183 | Kabardian-Balkar Republic | 7.1 | 9.4 | 2.4 | 33.41 |
| 1130 | Kamchatka kray | 3.4 | 4.5 | 1.1 | 33.40 |
| 1150 | Novosibirsk oblast | 35.4 | 47.2 | 11.8 | 33.35 |
| 1179 | Republic of Adygeya | 5.5 | 7.4 | 1.8 | 33.15 |
| 1157 | Perm kray | 33.3 | 44.3 | 11.0 | 33.11 |
| 1195 | Republic of Khakasia | 6.4 | 8.5 | 2.1 | 31.85 |
| 1182 | Republic of Dagestan | 15.1 | 19.8 | 4.7 | 31.15 |
| 1108 | Khabarovsk kray | 16.8 | 21.9 | 5.1 | 30.63 |
| 1181 | Republic of Buryatia | 10.4 | 13.5 | 3.2 | 30.51 |
| 1145 | Moscow | 132.5 | 172.8 | 40.3 | 30.39 |
| 1164 | Sakhalin oblast | 5.8 | 7.6 | 1.8 | 30.29 |
| 1176 | Zabaikalsk kray | 12.7 | 16.5 | 3.8 | 30.04 |
| 1132 | Kemerovo oblast | 36.9 | 47.6 | 10.7 | 29.04 |
| 1105 | Primorsky kray | 25.1 | 31.9 | 6.8 | 27.25 |

1. 2020

| ROSSTAT Code | | Region | | Expected deaths | | Observed deaths | | Excess deaths | | Excess death as percentage of expected in % | |
| --- | --- | --- | --- | --- | --- | --- | --- | --- | --- | --- | --- |
| 1196 | Chechen Republic | | 6.4 | | 9.4 | | 2.9 | | 45.97 | |  |
| 1182 | Republic of Dagestan | | 15.2 | | 19.8 | | 4.6 | | 30.21 | |  |
| 1192 | Republic of Tatarstan | | 42.3 | | 54.3 | | 12.0 | | 28.41 | |  |
| 1142 | Lipetzk oblast | | 15.8 | | 20.2 | | 4.4 | | 27.92 | |  |
| 1189 | Republic of Mordovia | | 10.3 | | 13.1 | | 2.8 | | 26.95 | |  |
| 1136 | Samara oblast | | 41.6 | | 52.9 | | 11.2 | | 26.91 | |  |
| 1197 | Chuvash Republic | | 14.8 | | 18.8 | | 4.0 | | 26.91 | |  |
| 1153 | Orenburg oblast | | 25.1 | | 31.8 | | 6.7 | | 26.72 | |  |
| 1156 | Penza oblast | | 18.1 | | 22.7 | | 4.6 | | 25.45 | |  |
| 1152 | Omsk oblast | | 24.0 | | 29.9 | | 5.9 | | 24.42 | |  |
| 1146 | Moscow oblast | | 90.0 | | 112.0 | | 22.0 | | 24.40 | |  |
| 1180 | Republic of Bashkortostan | | 48.6 | | 60.2 | | 11.7 | | 24.00 | |  |
| 1163 | Saratov oblast | | 32.9 | | 40.5 | | 7.6 | | 23.15 | |  |
| 1175 | Chelyabinsk oblast | | 44.9 | | 55.1 | | 10.2 | | 22.83 | |  |
| 1188 | Republic of Mariy El | | 8.0 | | 9.8 | | 1.8 | | 22.82 | |  |
| 1154 | Oryol oblast | | 10.9 | | 13.4 | | 2.5 | | 22.72 | |  |
| 1183 | Kabardian-Balkar Republic | | 7.2 | | 8.8 | | 1.6 | | 22.59 | |  |
| 1118 | Volgograd oblast | | 32.2 | | 39.4 | | 7.3 | | 22.57 | |  |
| 1173 | Ulyanovsk oblast | | 16.9 | | 20.7 | | 3.8 | | 22.53 | |  |
| 1161 | Ryazan oblast | | 16.4 | | 20.0 | | 3.6 | | 22.14 | |  |
| 1122 | Nizhny Novgorod oblast | | 45.4 | | 55.3 | | 9.9 | | 21.73 | |  |
| 1198 | Republic of Sakha (Yakutia) | | 7.5 | | 9.1 | | 1.6 | | 21.68 | |  |
| 1141 | Leningrad oblast | | 23.2 | | 28.1 | | 4.9 | | 21.10 | |  |
| 1129 | Kaluga oblast | | 14.3 | | 17.3 | | 3.0 | | 21.06 | |  |
| 1150 | Novosibirsk oblast | | 35.5 | | 42.8 | | 7.3 | | 20.62 | |  |
| 1194 | Udmurt Republic | | 17.6 | | 21.2 | | 3.6 | | 20.55 | |  |
| 1178 | Yaroslavl oblast | | 17.9 | | 21.5 | | 3.6 | | 20.42 | |  |
| 1147 | Murmansk oblast | | 8.3 | | 10.0 | | 1.7 | | 20.35 | |  |
| 1168 | Tambov oblast | | 14.9 | | 17.9 | | 3.0 | | 20.12 | |  |
| 1140 | Saint Petersburg | | 61.1 | | 73.3 | | 12.2 | | 19.90 | |  |
| 1108 | Khabarovsk kray | | 17.1 | | 20.4 | | 3.4 | | 19.89 | |  |
| 1169 | Tomsk oblast | | 12.0 | | 14.3 | | 2.3 | | 19.61 | |  |
| 1117 | Vladimir oblast | | 20.7 | | 24.7 | | 4.0 | | 19.29 | |  |
| 1114 | Belgorod oblast | | 20.2 | | 24.0 | | 3.9 | | 19.15 | |  |
| 1138 | Kursk oblast | | 16.3 | | 19.4 | | 3.1 | | 18.88 | |  |
| 1157 | Perm kray | | 33.8 | | 40.2 | | 6.4 | | 18.86 | |  |
| 1191 | Republic of Karachaevo-Chercassia | | 4.2 | | 5.0 | | .8 | | 18.74 | |  |
| 1133 | Kirov oblast | | 17.8 | | 21.1 | | 3.3 | | 18.66 | |  |
| 1120 | Voronezh oblast | | 32.2 | | 38.2 | | 6.0 | | 18.60 | |  |
| 1165 | Sverdlovsk oblast | | 57.0 | | 67.6 | | 10.6 | | 18.60 | |  |
| 1190 | Republic of North Ossetia - Alania | | 7.2 | | 8.6 | | 1.3 | | 18.57 | |  |
| 1160 | Rostov oblast | | 54.9 | | 64.8 | | 9.9 | | 18.07 | |  |
| 1112 | Astrakhan oblast | | 11.6 | | 13.6 | | 2.1 | | 18.07 | |  |
| 1101 | Altai kray | | 32.2 | | 38.0 | | 5.8 | | 17.93 | |  |
| 1110 | Amur oblast | | 10.8 | | 12.7 | | 1.9 | | 17.79 | |  |
| 1103 | Krasnodar kray | | 69.9 | | 82.3 | | 12.4 | | 17.70 | |  |
| 1115 | Bryansk oblast | | 17.1 | | 20.1 | | 3.0 | | 17.66 | |  |
| 1130 | Kamchatka kray | | 3.4 | | 4.0 | | .6 | | 17.41 | |  |
| 1186 | Republic of Karelia | | 8.7 | | 10.1 | | 1.5 | | 17.11 | |  |
| 1171001 | Tyumen Region (less AAs) | | 16.1 | | 18.8 | | 2.7 | | 17.07 | |  |
| 1158 | Pskov oblast | | 10.2 | | 11.9 | | 1.7 | | 16.75 | |  |
| 1134 | Kostroma oblast | | 9.0 | | 10.5 | | 1.5 | | 16.71 | |  |
| 1104 | Krasnoyarsk kray | | 35.1 | | 40.9 | | 5.8 | | 16.58 | |  |
| 1128 | Tver oblast | | 20.0 | | 23.2 | | 3.3 | | 16.40 | |  |
| 1107 | Stavropol kray | | 31.5 | | 36.6 | | 5.1 | | 16.27 | |  |
| 1166 | Smolensk oblast | | 13.7 | | 15.9 | | 2.2 | | 16.22 | |  |
| 1125 | Irkutsk oblast | | 31.0 | | 35.7 | | 4.7 | | 15.13 | |  |
| 1137 | Kurgan oblast | | 12.5 | | 14.3 | | 1.9 | | 14.96 | |  |
| 1127 | Kaliningrad oblast | | 11.7 | | 13.4 | | 1.7 | | 14.75 | |  |
| 1132 | Kemerovo oblast | | 37.5 | | 42.9 | | 5.5 | | 14.62 | |  |
| 1105 | Primorsky kray | | 25.4 | | 29.1 | | 3.7 | | 14.53 | |  |
| 1187 | Republic of Komi | | 9.7 | | 11.1 | | 1.4 | | 14.13 | |  |
| 1124 | Ivanovo oblast | | 15.4 | | 17.6 | | 2.2 | | 14.11 | |  |
| 1145 | Moscow | | 131.8 | | 150.2 | | 18.4 | | 13.97 | |  |
| 1119 | Vologda oblast | | 15.9 | | 18.1 | | 2.2 | | 13.80 | |  |
| 1149 | Novgorod oblast | | 9.6 | | 10.8 | | 1.3 | | 13.26 | |  |
| 1164 | Sakhalin oblast | | 5.9 | | 6.7 | | .8 | | 13.07 | |  |
| 1111001 | Arkhangelsk Region (less NAA) | | 14.5 | | 16.2 | | 1.7 | | 11.82 | |  |
| 1176 | Zabaikalsk kray | | 12.9 | | 14.4 | | 1.5 | | 11.58 | |  |
| 1181 | Republic of Buryatia | | 10.6 | | 11.8 | | 1.2 | | 11.26 | |  |
| 1179 | Republic of Adygeya | | 5.6 | | 6.2 | | .6 | | 10.37 | |  |
