# Supplementary material for: COVID-19 and excess mortality in Russia: Regional estimates of life expectancy losses in 2020 and excess deaths in 2021
Source: PLoS One. 2022 Nov 2;17(11):e0275967. doi: 10.1371/journal.pone.0275967 (PMC9629588; doi:10.1371/journal.pone.0275967)
Supplement: S2 Table — (DOCX) [file pone.0275967.s002.docx]

**S2 Table: Expected, observed and excess deaths (expressed in absolute and percentage terms) and life expectancy lost, regions of the Russian Federation with greater than 3,000 predicted deaths per year, 2020, urban and rural areas.**

1. URBAN

| Region | Excess deaths in thousands | Excess deaths as a percent of expected | Life expectancy loss |
| --- | --- | --- | --- |
| Lipetzk oblast | 3.04 | 32.6 | 2.9 |
| Republic of Mordovia | 1.73 | 31.1 | 2.4 |
| Chuvash Republic | 2.35 | 31.0 | 2.3 |
| Republic of Tatarstan | 9.06 | 30.8 | 2.5 |
| Orenburg oblast | 4.30 | 29.5 | 2.4 |
| Omsk oblast | 4.78 | 28.3 | 2.2 |
| Oryol oblast | 1.92 | 28.2 | 2.7 |
| Republic of Bashkortostan | 7.65 | 28.1 | 2.5 |
| Samara oblast | 9.06 | 27.5 | 2.3 |
| Penza oblast | 3.16 | 27.0 | 2.4 |
| Khanty-Mansi Autonomous Area – Yugra | 2.44 | 26.5 | 2.4 |
| Ryazan oblast | 2.89 | 26.2 | 2.5 |
| Moscow oblast | 18.83 | 25.5 | 2.3 |
| Chelyabinsk oblast | 9.09 | 25.2 | 2.0 |
| Saratov oblast | 6.09 | 25.1 | 2.1 |
| Kursk oblast | 2.39 | 24.8 | 2.2 |
| Tomsk oblast | 1.87 | 23.7 | 2.1 |
| Volgograd oblast | 5.72 | 23.5 | 2.2 |
| Altai kray | 3.97 | 23.4 | 1.8 |
| Nizhny Novgorod oblast | 8.07 | 23.2 | 2.1 |
| Republic of Dagestan | 1.39 | 23.2 | 2.6 |
| Republic of North Ossetia - Alania | 1.08 | 23.2 | 1.9 |
| Kaluga oblast | 2.48 | 22.8 | 2.0 |
| Perm kray | 5.57 | 22.6 | 2.1 |
| Udmurt Republic | 2.50 | 22.6 | 1.8 |
| Novosibirsk oblast | 6.03 | 22.4 | 2.0 |
| Ulyanovsk oblast | 2.72 | 22.3 | 1.8 |
| Tula oblast | 3.78 | 21.9 | 2.2 |
| Yaroslavl oblast | 3.11 | 21.8 | 1.9 |
| Republic of Karelia | 1.34 | 21.5 | 1.9 |
| Tambov oblast | 1.84 | 21.3 | 1.7 |
| The Russian Federation | 274.52 | 21.2 | 1.9 |
| Vladimir oblast | 3.38 | 21.2 | 2.0 |
| Kirov oblast | 2.65 | 21.0 | 1.7 |
| Kabardian-Balkar Republic | 0.81 | 21.0 | 2.1 |
| Belgorod oblast | 2.57 | 20.9 | 1.7 |
| Krasnoyarsk kray | 5.21 | 20.7 | 1.6 |
| Bryansk oblast | 2.37 | 20.7 | 1.9 |
| Voronezh oblast | 4.12 | 20.3 | 1.8 |
| Murmansk oblast | 1.60 | 20.3 | 2.0 |
| Kostroma oblast | 1.23 | 20.2 | 1.5 |
| Tver oblast | 2.92 | 20.1 | 1.7 |
| Leningrad oblast | 3.38 | 20.1 | 1.9 |
| Republic of Sakha (Yakutia) | 0.97 | 20.1 | 1.9 |
| Khabarovsk kray | 2.85 | 20.0 | 1.6 |
| Saint Petersburg city | 12.17 | 19.9 | 1.7 |
| Amur oblast | 1.40 | 19.8 | 1.6 |
| Republic of Mariy El | 1.01 | 19.7 | 1.4 |
| Sverdlovsk oblast | 9.13 | 19.2 | 1.5 |
| Tyumen Region less autonomous areas | 1.81 | 19.1 | 1.6 |
| Rostov oblast | 6.97 | 19.0 | 1.7 |
| Pskov oblast | 1.30 | 18.8 | 1.2 |
| Kurgan oblast | 1.36 | 18.6 | 1.4 |
| Krasnodar kray | 7.19 | 18.5 | 1.5 |
| Stavropol kray | 3.25 | 18.3 | 1.7 |
| Smolensk oblast | 1.70 | 18.3 | 1.7 |
| Irkutsk oblast | 4.30 | 17.9 | 1.6 |
| Republic of Komi | 1.21 | 17.9 | 1.2 |
| Republic of Khakasia | 0.73 | 17.1 | 1.2 |
| Arkhangelsk Region less autonomous area | 1.69 | 17.0 | 1.2 |
| Novgorod oblast | 1.08 | 16.9 | 1.0 |
| Kemerovo oblast | 5.18 | 16.3 | 1.5 |
| Zabaikalsk kray | 1.31 | 16.1 | 1.2 |
| Primorsky kray | 2.99 | 15.9 | 1.3 |
| Astrakhan oblast | 1.25 | 15.5 | 1.4 |
| Ivanovo oblast | 1.89 | 15.4 | 1.1 |
| Vologda oblast | 1.60 | 15.3 | 1.0 |
| Republic of Buryatia | 0.86 | 15.2 | 1.0 |
| Sakhalin oblast | 0.67 | 14.5 | 1.0 |
| Moscow city | 17.72 | 13.7 | 0.9 |
| Kaliningrad oblast | 1.27 | 13.6 | 0.6 |
|  |  |  |  |
| **The Russian Federation** | **274.52** | **21.2** | **1.9** |

1. RURAL

| Region | Excess deaths in thousands | Excess deaths as a percent of expected | Life expectancy loss |
| --- | --- | --- | --- |
| Republic of Dagestan | 3.19 | 34.8 | 3.5 |
| Samara oblast | 2.15 | 24.7 | 2.9 |
| Kabardian-Balkar Republic | 0.81 | 24.5 | 2.6 |
| Astrakhan oblast | 0.84 | 23.8 | 2.4 |
| Leningrad oblast | 1.51 | 23.7 | 3.6 |
| Ulyanovsk oblast | 1.09 | 23.1 | 3.0 |
| Republic of Tatarstan | 2.96 | 23.0 | 2.7 |
| Orenburg oblast | 2.41 | 22.9 | 2.9 |
| Penza oblast | 1.44 | 22.6 | 2.5 |
| Chuvash Republic | 1.65 | 22.6 | 3.5 |
| Republic of Mordovia | 1.05 | 22.1 | 2.9 |
| Lipetzk oblast | 1.36 | 21.2 | 3.0 |
| Volgograd oblast | 1.54 | 19.8 | 2.5 |
| Moscow oblast | 3.13 | 19.4 | 2.5 |
| Republic of Bashkortostan | 4.01 | 18.8 | 2.6 |
| Tambov oblast | 1.15 | 18.5 | 2.4 |
| Saratov oblast | 1.53 | 17.7 | 2.0 |
| Nizhny Novgorod oblast | 1.80 | 17.0 | 2.5 |
| Udmurt Republic | 1.11 | 17.0 | 2.3 |
| Krasnodar kray | 5.18 | 16.7 | 2.1 |
| Belgorod oblast | 1.30 | 16.4 | 2.3 |
| Rostov oblast | 2.95 | 16.2 | 2.0 |
| Voronezh oblast | 1.88 | 15.7 | 2.0 |
| Kaluga oblast | 0.53 | 15.6 | 2.7 |
| The Russian Federation | 76.64 | 15.5 | 2.0 |
| Sverdlovsk oblast | 1.48 | 15.5 | 2.3 |
| Omsk oblast | 1.09 | 15.3 | 1.8 |
| Novosibirsk oblast | 1.29 | 15.0 | 2.1 |
| Yaroslavl oblast | 0.54 | 14.9 | 2.0 |
| Tyumen Region less autonomous areas | 0.94 | 14.1 | 1.8 |
| Amur oblast | 0.52 | 13.9 | 1.9 |
| Ryazan oblast | 0.74 | 13.8 | 2.5 |
| Stavropol kray | 1.87 | 13.7 | 1.4 |
| Oryol oblast | 0.57 | 13.7 | 2.1 |
| Chelyabinsk oblast | 1.15 | 13.2 | 1.5 |
| Vladimir oblast | 0.61 | 12.9 | 1.8 |
| Kirov oblast | 0.67 | 12.9 | 2.5 |
| Tula oblast | 0.72 | 12.9 | 2.0 |
| Pskov oblast | 0.40 | 12.4 | 3.4 |
| Altai kray | 1.82 | 11.9 | 1.3 |
| Smolensk oblast | 0.52 | 11.8 | 2.8 |
| Tomsk oblast | 0.47 | 11.7 | 1.5 |
| Bryansk oblast | 0.65 | 11.5 | 1.9 |
| Vologda oblast | 0.60 | 11.0 | 2.0 |
| Primorsky kray | 0.70 | 10.6 | 1.5 |
| Kursk oblast | 0.70 | 10.4 | 1.2 |
| Kurgan oblast | 0.51 | 9.8 | 1.7 |
| Ivanovo oblast | 0.28 | 9.0 | 1.8 |
| Perm kray | 0.81 | 8.9 | 1.5 |
| Republic of Buryatia | 0.33 | 6.7 | 0.8 |
| Tver oblast | 0.35 | 6.5 | 1.9 |
| Republic of Adygeya | 0.20 | 6.4 | 0.7 |
| Krasnoyarsk kray | 0.61 | 6.1 | 1.0 |
| Novgorod oblast | 0.19 | 6.0 | 1.2 |
| Irkutsk oblast | 0.39 | 5.6 | 1.3 |
| Kemerovo oblast | 0.29 | 5.3 | 1.4 |
| Zabaikalsk kray | 0.19 | 4.0 | 1.1 |
| Arkhangelsk Region less autonomous area | 0.02 | 0.4 | 0.6 |
| Chechen Republic | -0.01 | -0.4 | -0.1 |
|  |  |  |  |
|  |  |  |  |
| **The Russian Federation** | **76.64** | **15.5** | **2.0** |
