# Supplementary material for: COVID-19 and excess mortality in Russia: Regional estimates of life expectancy losses in 2020 and excess deaths in 2021
Source: PLoS One. 2022 Nov 2;17(11):e0275967. doi: 10.1371/journal.pone.0275967 (PMC9629588; doi:10.1371/journal.pone.0275967)
Supplement: S3 Table — (DOCX) [file pone.0275967.s003.docx]

**S3 Table: Expected, observed and excess deaths (expressed in absolute and percentage terms), regions of the Russian Federation with greater than 3,000 predicted deaths per year, 2020, males and females.**

1. **MALES**

| Region | Excess deaths in thousands | Excess deaths as a percent of expected | Life expectancy loss |
| --- | --- | --- | --- |
| Chechen Republic | 1.63 | 48.2 | 4.7 |
| Republic of Dagestan | 2.63 | 33.1 | 3.9 |
| Lipetzk oblast | 2.17 | 28.0 | 3.3 |
| Republic of Tatarstan | 5.93 | 27.7 | 3.0 |
| Moscow oblast | 11.07 | 25.7 | 2.9 |
| Oryol oblast | 1.34 | 25.7 | 2.8 |
| Republic of Mordovia | 1.31 | 25.7 | 2.5 |
| Chuvash Republic | 1.98 | 25.5 | 2.7 |
| Samara oblast | 5.24 | 25.4 | 2.7 |
| Kabardian-Balkar Republic | 0.91 | 25.4 | 3.1 |
| Omsk oblast | 2.85 | 23.5 | 2.1 |
| Penza oblast | 2.10 | 23.5 | 2.5 |
| Orenburg oblast | 3.01 | 23.4 | 2.4 |
| Volgograd oblast | 3.57 | 22.6 | 2.5 |
| Republic of Bashkortostan | 5.71 | 22.6 | 2.5 |
| Novosibirsk oblast | 3.97 | 22.4 | 2.3 |
| Ulyanovsk oblast | 1.85 | 22.2 | 2.3 |
| Chelyabinsk oblast | 4.99 | 22.2 | 2.0 |
| Republic of Sakha (Yakutia) | 0.96 | 22.1 | 2.8 |
| Ryazan oblast | 1.73 | 22.0 | 2.6 |
| Murmansk oblast | 0.92 | 21.8 | 2.6 |
| Republic of Mariy El | 0.91 | 21.7 | 2.3 |
| Saint Petersburg city | 6.02 | 21.6 | 2.5 |
| Khanty-Mansi Autonomous Area – Yugra | 1.31 | 21.6 | 2.4 |
| Saratov oblast | 3.48 | 21.5 | 2.2 |
| Yaroslavl oblast | 1.78 | 21.3 | 2.5 |
| Nizhny Novgorod oblast | 4.61 | 21.0 | 2.4 |
| Leningrad oblast | 2.41 | 20.8 | 2.8 |
| Belgorod oblast | 2.00 | 20.1 | 2.2 |
| Tomsk oblast | 1.27 | 20.1 | 2.2 |
| Tula oblast | 2.15 | 20.1 | 2.4 |
| Voronezh oblast | 3.11 | 19.8 | 2.3 |
| Udmurt Republic | 1.79 | 19.8 | 2.3 |
| Rostov oblast | 5.20 | 19.5 | 2.2 |
| Kaluga oblast | 1.40 | 19.4 | 2.2 |
| Kursk oblast | 1.54 | 19.3 | 2.0 |
| The Russian Federation | 171.60 | 19.2 | 2.2 |
| Khabarovsk kray | 1.71 | 18.7 | 1.8 |
| Pskov oblast | 0.90 | 18.7 | 2.2 |
| Republic of Karelia | 0.81 | 18.6 | 2.4 |
| Stavropol kray | 2.79 | 18.0 | 2.0 |
| Vladimir oblast | 1.78 | 18.0 | 2.1 |
| Tver oblast | 1.71 | 18.0 | 2.1 |
| Altai kray | 2.92 | 17.8 | 1.5 |
| Krasnodar kray | 6.27 | 17.7 | 2.0 |
| Amur oblast | 1.01 | 17.7 | 1.9 |
| Tambov oblast | 1.29 | 17.7 | 1.9 |
| Republic of North Ossetia - Alania | 0.65 | 17.7 | 2.0 |
| Perm kray | 3.01 | 17.4 | 2.0 |
| Smolensk oblast | 1.16 | 17.3 | 2.4 |
| Tyumen Region less autonomous areas | 1.47 | 17.3 | 1.9 |
| Krasnoyarsk kray | 3.10 | 17.0 | 1.8 |
| Sverdlovsk oblast | 4.86 | 16.9 | 1.7 |
| Kirov oblast | 1.49 | 16.8 | 1.8 |
| Bryansk oblast | 1.37 | 16.3 | 2.0 |
| Astrakhan oblast | 0.93 | 16.0 | 1.7 |
| Ivanovo oblast | 1.11 | 15.7 | 1.9 |
| Kemerovo oblast | 2.96 | 15.6 | 1.7 |
| Novgorod oblast | 0.70 | 15.1 | 1.4 |
| Republic of Komi | 0.77 | 15.1 | 1.4 |
| Kostroma oblast | 0.66 | 15.0 | 1.7 |
| Kurgan oblast | 0.87 | 13.9 | 1.2 |
| Irkutsk oblast | 2.22 | 13.7 | 1.7 |
| Primorsky kray | 1.81 | 13.5 | 1.5 |
| Republic of Khakasia | 0.46 | 13.2 | 1.2 |
| Vologda oblast | 1.05 | 12.9 | 1.5 |
| Arkhangelsk Region less autonomous area | 0.95 | 12.8 | 1.3 |
| Kaliningrad oblast | 0.70 | 12.3 | 0.9 |
| Sakhalin oblast | 0.39 | 12.1 | 1.0 |
| Moscow city | 6.53 | 10.1 | 0.7 |
| Republic of Buryatia | 0.58 | 10.1 | 0.7 |
| Zabaikalsk kray | 0.71 | 9.9 | 0.9 |
|  |  |  |  |
|  |  |  |  |
|  |  |  |  |
| **The Russian Federation** | **171.60** | **19.2** | **2.2** |

1. **FEMALES**

| Region | Excess deaths in thousands | Excess deaths as a percent of expected | Life expectancy loss |
| --- | --- | --- | --- |
| Chechen Republic | 1.32 | 43.5 | 3.8 |
| Khanty-Mansi Autonomous Area – Yugra | 1.36 | 32.9 | 2.7 |
| Orenburg oblast | 3.69 | 30.2 | 2.9 |
| Republic of Tatarstan | 6.08 | 29.1 | 2.5 |
| Samara oblast | 5.96 | 28.4 | 2.7 |
| Chuvash Republic | 2.02 | 28.4 | 2.7 |
| Republic of Mordovia | 1.47 | 28.2 | 2.7 |
| Lipetzk oblast | 2.24 | 27.8 | 2.7 |
| Penza oblast | 2.51 | 27.4 | 2.7 |
| Republic of Dagestan | 1.95 | 27.1 | 2.5 |
| Republic of Bashkortostan | 5.95 | 25.5 | 2.7 |
| Omsk oblast | 3.02 | 25.4 | 2.4 |
| Saratov oblast | 4.14 | 24.8 | 2.5 |
| Republic of Mariy El | 0.90 | 24.0 | 2.2 |
| Chelyabinsk oblast | 5.25 | 23.5 | 2.2 |
| Moscow oblast | 10.90 | 23.2 | 2.2 |
| Ulyanovsk oblast | 1.96 | 22.9 | 2.2 |
| Kaluga oblast | 1.61 | 22.7 | 2.4 |
| Volgograd oblast | 3.69 | 22.6 | 2.4 |
| Tambov oblast | 1.70 | 22.5 | 2.3 |
| Nizhny Novgorod oblast | 5.27 | 22.4 | 2.3 |
| Ryazan oblast | 1.89 | 22.3 | 2.8 |
| Leningrad oblast | 2.49 | 21.4 | 2.2 |
| Khabarovsk kray | 1.68 | 21.3 | 1.9 |
| Udmurt Republic | 1.82 | 21.3 | 2.0 |
| Republic of Sakha (Yakutia) | 0.66 | 21.1 | 2.1 |
| Kirov oblast | 1.83 | 20.6 | 1.8 |
| Vladimir oblast | 2.21 | 20.5 | 2.3 |
| Perm kray | 3.37 | 20.4 | 2.2 |
| Sverdlovsk oblast | 5.75 | 20.3 | 2.0 |
| The Russian Federation | 179.56 | 20.1 | 2.0 |
| Astrakhan oblast | 1.16 | 20.1 | 2.1 |
| Oryol oblast | 1.14 | 20.0 | 2.5 |
| Kabardian-Balkar Republic | 0.70 | 19.8 | 2.0 |
| Yaroslavl oblast | 1.87 | 19.7 | 2.0 |
| Republic of North Ossetia - Alania | 0.69 | 19.5 | 1.9 |
| Tula oblast | 2.34 | 19.4 | 2.4 |
| Tomsk oblast | 1.08 | 19.1 | 1.8 |
| Bryansk oblast | 1.65 | 19.0 | 2.1 |
| Novosibirsk oblast | 3.35 | 18.9 | 2.0 |
| Murmansk oblast | 0.76 | 18.8 | 1.6 |
| Kursk oblast | 1.55 | 18.5 | 2.0 |
| Saint Petersburg city | 6.14 | 18.5 | 2.2 |
| Kostroma oblast | 0.84 | 18.4 | 1.8 |
| Belgorod oblast | 1.86 | 18.2 | 1.9 |
| Altai kray | 2.86 | 18.1 | 1.8 |
| Amur oblast | 0.91 | 17.9 | 1.4 |
| Krasnodar kray | 6.10 | 17.7 | 1.9 |
| Moscow city | 11.88 | 17.7 | 1.6 |
| Voronezh oblast | 2.88 | 17.5 | 1.8 |
| Kaliningrad oblast | 1.02 | 17.1 | 1.3 |
| Republic of Khakasia | 0.51 | 16.8 | 1.5 |
| Tyumen Region less autonomous areas | 1.27 | 16.8 | 1.6 |
| Rostov oblast | 4.71 | 16.7 | 1.8 |
| Irkutsk oblast | 2.47 | 16.6 | 1.6 |
| Krasnoyarsk kray | 2.72 | 16.1 | 1.4 |
| Kurgan oblast | 0.99 | 16.1 | 1.8 |
| Primorsky kray | 1.88 | 15.6 | 1.2 |
| Republic of Karelia | 0.68 | 15.6 | 1.4 |
| Smolensk oblast | 1.06 | 15.2 | 1.7 |
| Pskov oblast | 0.80 | 15.0 | 1.7 |
| Tver oblast | 1.56 | 14.9 | 1.6 |
| Vologda oblast | 1.14 | 14.8 | 1.3 |
| Stavropol kray | 2.33 | 14.6 | 1.5 |
| Kemerovo oblast | 2.52 | 13.6 | 1.6 |
| Zabaikalsk kray | 0.79 | 13.6 | 1.2 |
| Republic of Komi | 0.60 | 13.1 | 1.0 |
| Ivanovo oblast | 1.06 | 12.8 | 1.1 |
| Republic of Buryatia | 0.62 | 12.6 | 1.4 |
| Novgorod oblast | 0.57 | 11.5 | 1.2 |
| Arkhangelsk Region less autonomous area | 0.76 | 10.8 | 0.7 |
|  |  |  |  |
|  |  |  |  |
| **The Russian Federation** | **179.56** | **20.1** | **2.0** |
