# Supplementary material for: COVID-19 and excess mortality in Russia: Regional estimates of life expectancy losses in 2020 and excess deaths in 2021
Source: PLoS One. 2022 Nov 2;17(11):e0275967. doi: 10.1371/journal.pone.0275967 (PMC9629588; doi:10.1371/journal.pone.0275967)
Supplement: S4 Table — (DOCX) [file pone.0275967.s004.docx]

**S4 Table: Expected, observed and excess deaths (expressed in absolute and percentage terms), regions of the Russian Federation with greater than 3,000 predicted deaths per year, 2020, aged below and above 65.**

1. Below 65

| Region | Excess deaths in thousands | Excess deaths as a percent of expected | Share of excess death below 65 in all excess death in % |
| --- | --- | --- | --- |
| Lipetzk oblast | 1.41 | 30.5 | 32.1 |
| Oryol oblast | 0.86 | 27.0 | 34.7 |
| Republic of Mordovia | 0.78 | 24.6 | 28.1 |
| Ryazan oblast | 1.12 | 24.3 | 30.9 |
| Republic of Dagestan | 1.29 | 24.2 | 28.2 |
| Moscow oblast | 6.63 | 23.2 | 30.2 |
| Chuvash Republic | 1.14 | 22.5 | 28.5 |
| Khanty-Mansi Autonomous Area – Yugra | 1.20 | 22.3 | 44.9 |
| Penza oblast | 1.14 | 21.5 | 24.7 |
| Volgograd oblast | 1.95 | 21.1 | 26.9 |
| Republic of Tatarstan | 2.80 | 21.0 | 23.3 |
| Orenburg oblast | 1.80 | 20.6 | 26.9 |
| Nizhny Novgorod oblast | 2.91 | 20.5 | 29.5 |
| Vladimir oblast | 1.29 | 20.4 | 32.3 |
| Samara oblast | 2.73 | 20.2 | 24.3 |
| Pskov oblast | 0.65 | 20.0 | 38.0 |
| Republic of Karelia | 0.61 | 19.8 | 41.3 |
| Saratov oblast | 1.95 | 19.7 | 25.7 |
| Republic of Bashkortostan | 3.41 | 19.6 | 29.3 |
| Tula oblast | 1.31 | 19.3 | 29.2 |
| Tambov oblast | 0.81 | 19.0 | 27.1 |
| Yaroslavl oblast | 1.00 | 18.7 | 27.4 |
| Belgorod oblast | 1.08 | 18.6 | 27.9 |
| Leningrad oblast | 1.48 | 18.4 | 30.2 |
| Voronezh oblast | 1.69 | 18.2 | 28.2 |
| Kaluga oblast | 0.85 | 18.2 | 28.2 |
| Tver oblast | 1.13 | 18.1 | 34.6 |
| Ulyanovsk oblast | 0.93 | 17.6 | 24.4 |
| Republic of Sakha (Yakutia) | 0.64 | 17.3 | 39.7 |
| Bryansk oblast | 0.96 | 17.2 | 31.7 |
| Kursk oblast | 0.87 | 17.1 | 28.2 |
| Murmansk oblast | 0.59 | 16.9 | 35.2 |
| Stavropol kray | 1.50 | 16.5 | 29.3 |
| Novosibirsk oblast | 1.99 | 16.4 | 27.2 |
| Omsk oblast | 1.35 | 16.3 | 23.0 |
| Krasnodar kray | 3.44 | 16.2 | 27.8 |
| Amur oblast | 0.70 | 16.2 | 36.7 |
| Udmurt Republic | 1.01 | 16.2 | 28.0 |
| The Russian Federation | 94.09 | 15.9 | 26.8 |
| Astrakhan oblast | 0.60 | 15.8 | 28.9 |
| Saint Petersburg city | 2.74 | 15.5 | 22.5 |
| Rostov oblast | 2.46 | 15.4 | 24.8 |
| Smolensk oblast | 0.70 | 15.4 | 31.7 |
| Sverdlovsk oblast | 2.92 | 15.1 | 27.5 |
| Perm kray | 1.80 | 14.7 | 28.1 |
| Chelyabinsk oblast | 2.18 | 14.4 | 21.3 |
| Tyumen Region less autonomous areas | 0.87 | 14.3 | 31.7 |
| Tomsk oblast | 0.61 | 13.6 | 25.8 |
| Ivanovo oblast | 0.61 | 13.1 | 28.1 |
| Khabarovsk kray | 0.86 | 12.9 | 25.2 |
| Kirov oblast | 0.70 | 12.7 | 21.2 |
| Novgorod oblast | 0.39 | 12.3 | 30.7 |
| Krasnoyarsk kray | 1.65 | 12.2 | 28.3 |
| Vologda oblast | 0.66 | 12.0 | 30.0 |
| Irkutsk oblast | 1.49 | 12.0 | 31.9 |
| Kemerovo oblast | 1.69 | 11.7 | 30.8 |
| Kurgan oblast | 0.45 | 11.3 | 24.3 |
| Altai kray | 1.18 | 10.8 | 20.5 |
| Primorsky kray | 0.99 | 10.6 | 26.9 |
| Republic of Komi | 0.37 | 8.7 | 26.8 |
| Arkhangelsk Region less autonomous area | 0.44 | 8.5 | 25.6 |
| Kaliningrad oblast | 0.28 | 7.3 | 16.5 |
| Zabaikalsk kray | 0.34 | 5.9 | 22.4 |
| Republic of Buryatia | 0.24 | 5.2 | 19.7 |
| Moscow city | 2.00 | 5.1 | 10.9 |
|  |  |  |  |
| **The Russian Federation** | **94.09** | **15.9** | **26.8** |

1. Above 65

| Region | Excess deaths in thousands | Excess deaths as a percent of expected | Share of excess death 65+ in all excess death in % |
| --- | --- | --- | --- |
| Chechen Republic | 2.04 | 55.9 | 69.2 |
| Republic of Dagestan | 3.29 | 33.5 | 71.8 |
| Republic of Tatarstan | 9.22 | 31.8 | 76.7 |
| Khanty-Mansi Autonomous Area – Yugra | 1.47 | 30.5 | 55.1 |
| Samara oblast | 8.48 | 30.2 | 75.7 |
| Orenburg oblast | 4.90 | 30.0 | 73.1 |
| Chuvash Republic | 2.86 | 29.2 | 71.5 |
| Omsk oblast | 4.52 | 28.7 | 77.0 |
| Republic of Mariy El | 1.42 | 28.0 | 78.4 |
| Republic of Mordovia | 2.00 | 28.0 | 71.9 |
| Chelyabinsk oblast | 8.06 | 27.2 | 78.7 |
| Penza oblast | 3.47 | 27.1 | 75.3 |
| Lipetzk oblast | 2.99 | 26.8 | 67.9 |
| Republic of Bashkortostan | 8.24 | 26.5 | 70.7 |
| Republic of Sakha (Yakutia) | 0.98 | 26.1 | 60.3 |
| Moscow oblast | 15.33 | 25.0 | 69.8 |
| Ulyanovsk oblast | 2.88 | 24.8 | 75.6 |
| Saratov oblast | 5.66 | 24.6 | 74.3 |
| Khabarovsk kray | 2.54 | 24.4 | 74.8 |
| Volgograd oblast | 5.30 | 23.2 | 73.1 |
| Tomsk oblast | 1.74 | 23.2 | 74.2 |
| Udmurt Republic | 2.60 | 23.0 | 72.0 |
| Murmansk oblast | 1.09 | 22.9 | 64.8 |
| Novosibirsk oblast | 5.33 | 22.8 | 72.8 |
| Leningrad oblast | 3.42 | 22.6 | 69.8 |
| Kaluga oblast | 2.16 | 22.4 | 71.8 |
| Kabardian-Balkar Republic | 1.11 | 22.4 | 68.4 |
| Nizhny Novgorod oblast | 6.96 | 22.3 | 70.5 |
| Saint Petersburg city | 9.42 | 21.7 | 77.5 |
| Altai kray | 4.60 | 21.6 | 79.5 |
| The Russian Federation | 257.07 | 21.5 | 73.2 |
| Kirov oblast | 2.62 | 21.4 | 78.8 |
| Ryazan oblast | 2.50 | 21.3 | 69.1 |
| Perm kray | 4.59 | 21.2 | 71.9 |
| Yaroslavl oblast | 2.65 | 21.1 | 72.6 |
| Oryol oblast | 1.62 | 21.0 | 65.3 |
| Tambov oblast | 2.18 | 20.5 | 72.9 |
| Sverdlovsk oblast | 7.69 | 20.4 | 72.5 |
| Tula oblast | 3.19 | 19.9 | 70.8 |
| Kursk oblast | 2.21 | 19.7 | 71.8 |
| Kostroma oblast | 1.20 | 19.4 | 79.7 |
| Krasnoyarsk kray | 4.17 | 19.3 | 71.7 |
| Belgorod oblast | 2.79 | 19.3 | 72.1 |
| Astrakhan oblast | 1.49 | 19.2 | 71.1 |
| Rostov oblast | 7.45 | 19.2 | 75.2 |
| Amur oblast | 1.22 | 18.9 | 63.3 |
| Vladimir oblast | 2.70 | 18.8 | 67.7 |
| Voronezh oblast | 4.30 | 18.8 | 71.8 |
| Tyumen Region less autonomous areas | 1.87 | 18.8 | 68.3 |
| Republic of North Ossetia - Alania | 0.97 | 18.6 | 72.3 |
| Kaliningrad oblast | 1.44 | 18.5 | 83.5 |
| Krasnodar kray | 8.93 | 18.3 | 72.2 |
| Republic of Komi | 1.00 | 18.2 | 73.2 |
| Bryansk oblast | 2.07 | 17.9 | 68.3 |
| Moscow city | 16.40 | 17.8 | 89.1 |
| Republic of Khakasia | 0.69 | 17.4 | 71.0 |
| Irkutsk oblast | 3.20 | 17.2 | 68.1 |
| Sakhalin oblast | 0.59 | 17.1 | 76.3 |
| Primorsky kray | 2.70 | 16.8 | 73.1 |
| Kurgan oblast | 1.41 | 16.7 | 75.7 |
| Smolensk oblast | 1.52 | 16.7 | 68.3 |
| Kemerovo oblast | 3.79 | 16.5 | 69.2 |
| Stavropol kray | 3.62 | 16.2 | 70.7 |
| Zabaikalsk kray | 1.16 | 16.1 | 77.6 |
| Republic of Buryatia | 0.96 | 15.7 | 80.3 |
| Tver oblast | 2.14 | 15.6 | 65.4 |
| Republic of Karelia | 0.87 | 15.6 | 58.7 |
| Pskov oblast | 1.06 | 15.2 | 62.0 |
| Vologda oblast | 1.54 | 14.7 | 70.0 |
| Ivanovo oblast | 1.56 | 14.6 | 71.9 |
| Novgorod oblast | 0.88 | 13.8 | 69.3 |
| Arkhangelsk Region less autonomous area | 1.27 | 13.6 | 74.4 |
| Republic of Adygeya | 0.45 | 11.4 | 77.6 |
| **The Russian Federation** | **257.07** | **21.5** | **73.2** |
