# Supplementary material for: COVID-19 and excess mortality in Russia: Regional estimates of life expectancy losses in 2020 and excess deaths in 2021
Source: PLoS One. 2022 Nov 2;17(11):e0275967. doi: 10.1371/journal.pone.0275967 (PMC9629588; doi:10.1371/journal.pone.0275967)
Supplement: S5 Table — (DOCX) [file pone.0275967.s005.docx]

**S5 Table: Remaining life expectancy in regions of the Russian Federation with greater than 3,000 predicted deaths per year, 2020, males and females.**

1. **Males**

| Region | Excess deaths as a percent of expected | RLED |
| --- | --- | --- |
| Republic of Sakha (Yakutia) | 22.5 | 18.4 |
| Khanty-Mansi Autonomous Area – Yugra | 22.6 | 17.2 |
| Kabardian-Balkar Republic | 25.8 | 17.1 |
| Republic of Dagestan | 34.4 | 16.8 |
| Leningrad oblast | 20.9 | 16.5 |
| Murmansk oblast | 22.3 | 16.3 |
| Republic of Karelia | 18.7 | 15.8 |
| Republic of North Ossetia - Alania | 18.9 | 15.7 |
| Smolensk oblast | 17.3 | 15.4 |
| Lipetzk oblast | 28.2 | 14.9 |
| Bryansk oblast | 16.6 | 14.8 |
| Chechen Republic | 50.9 | 14.8 |
| Astrakhan oblast | 16.5 | 14.7 |
| Moscow oblast | 26.0 | 14.7 |
| Vologda oblast | 13.1 | 14.6 |
| Pskov oblast | 19.0 | 14.6 |
| Saint Petersburg city | 21.9 | 14.6 |
| Vladimir oblast | 18.3 | 14.5 |
| Udmurt Republic | 20.0 | 14.5 |
| Irkutsk oblast | 13.9 | 14.4 |
| Tyumen Region less autonomous areas | 17.6 | 14.4 |
| Stavropol kray | 18.4 | 14.3 |
| Chuvash Republic | 25.9 | 14.3 |
| Tomsk oblast | 20.5 | 14.2 |
| Ivanovo oblast | 15.8 | 14.1 |
| Tambov oblast | 18.0 | 14.1 |
| Republic of Mariy El | 22.1 | 14.1 |
| Voronezh oblast | 20.0 | 14.0 |
| Tver oblast | 18.3 | 14.0 |
| Kaluga oblast | 19.7 | 14.0 |
| Yaroslavl oblast | 21.4 | 14.0 |
| Ryazan oblast | 22.2 | 13.9 |
| Tula oblast | 20.3 | 13.9 |
| Republic of Komi | 15.6 | 13.9 |
| The Russian Federation | 19.5 | 13.8 |
| Krasnodar kray | 17.9 | 13.8 |
| Amur oblast | 18.0 | 13.8 |
| Volgograd oblast | 22.7 | 13.8 |
| Nizhny Novgorod oblast | 21.2 | 13.8 |
| Kostroma oblast | 15.4 | 13.8 |
| Moscow city | 10.8 | 13.7 |
| Republic of Mordovia | 26.1 | 13.7 |
| Arkhangelsk Region less autonomous area | 13.2 | 13.7 |
| Oryol oblast | 26.0 | 13.5 |
| Republic of Tatarstan | 28.0 | 13.5 |
| Primorsky kray | 13.8 | 13.4 |
| Belgorod oblast | 20.2 | 13.4 |
| Sakhalin oblast | 12.8 | 13.4 |
| Krasnoyarsk kray | 17.4 | 13.3 |
| Kemerovo oblast | 15.9 | 13.3 |
| Penza oblast | 23.7 | 13.3 |
| Rostov oblast | 19.5 | 13.3 |
| Ulyanovsk oblast | 22.5 | 13.3 |
| Republic of Bashkortostan | 22.9 | 13.3 |
| Republic of Khakasia | 13.7 | 13.3 |
| Kursk oblast | 19.6 | 13.2 |
| Perm kray | 17.6 | 13.2 |
| Zabaikalsk kray | 10.3 | 13.2 |
| Samara oblast | 25.6 | 13.1 |
| Novgorod oblast | 15.6 | 13.1 |
| Novosibirsk oblast | 22.6 | 12.9 |
| Saratov oblast | 21.8 | 12.9 |
| Sverdlovsk oblast | 17.3 | 12.9 |
| Orenburg oblast | 23.7 | 12.8 |
| Kirov oblast | 16.7 | 12.7 |
| Kaliningrad oblast | 12.7 | 12.5 |
| Kurgan oblast | 14.0 | 12.5 |
| Khabarovsk kray | 19.2 | 12.4 |
| Omsk oblast | 24.1 | 11.8 |
| Chelyabinsk oblast | 22.5 | 11.8 |
| Republic of Buryatia | 10.7 | 11.7 |
| Altai kray | 18.0 | 10.9 |
|  |  |  |
| **The Russian Federation** | **19.5** | **13.8** |

1. Females

| Region | Excess deaths as a percent of expected | RLED |
| --- | --- | --- |
| Republic of Sakha (Yakutia) | 21.8 | 17.9 |
| Republic of Buryatia | 12.7 | 16.3 |
| Khanty-Mansi Autonomous Area – Yugra | 33.9 | 16.3 |
| Republic of Dagestan | 28.2 | 16.0 |
| Kabardian-Balkar Republic | 20.1 | 15.3 |
| Republic of Karelia | 16.3 | 15.3 |
| Republic of Komi | 13.9 | 15.2 |
| Kemerovo oblast | 13.7 | 15.0 |
| Zabaikalsk kray | 14.1 | 15.0 |
| Republic of Khakasia | 17.4 | 15.0 |
| Republic of North Ossetia - Alania | 20.2 | 14.9 |
| Chechen Republic | 46.3 | 14.9 |
| Ryazan oblast | 22.4 | 14.7 |
| Oryol oblast | 20.1 | 14.5 |
| Tula oblast | 19.5 | 14.5 |
| Stavropol kray | 15.0 | 14.3 |
| Pskov oblast | 15.2 | 14.2 |
| Smolensk oblast | 15.4 | 14.2 |
| Amur oblast | 18.8 | 14.1 |
| Kurgan oblast | 16.2 | 14.1 |
| Novgorod oblast | 11.8 | 14.1 |
| Tyumen Region less autonomous areas | 17.1 | 14.1 |
| Astrakhan oblast | 20.2 | 14.0 |
| Bryansk oblast | 19.2 | 14.0 |
| Republic of Bashkortostan | 25.9 | 13.9 |
| Leningrad oblast | 21.6 | 13.8 |
| Tver oblast | 15.2 | 13.7 |
| Murmansk oblast | 19.4 | 13.7 |
| Saint Petersburg city | 18.6 | 13.7 |
| Kaluga oblast | 23.1 | 13.6 |
| Belgorod oblast | 18.4 | 13.5 |
| Republic of Mariy El | 24.2 | 13.5 |
| Perm kray | 20.5 | 13.4 |
| Republic of Mordovia | 28.5 | 13.4 |
| Arkhangelsk Region less autonomous area | 11.5 | 13.4 |
| Krasnodar kray | 17.8 | 13.3 |
| Volgograd oblast | 22.7 | 13.3 |
| Vologda oblast | 15.2 | 13.3 |
| Tambov oblast | 22.7 | 13.3 |
| Tomsk oblast | 19.4 | 13.3 |
| Altai kray | 18.4 | 13.1 |
| Kursk oblast | 18.8 | 13.1 |
| Irkutsk oblast | 16.9 | 13.0 |
| Rostov oblast | 16.8 | 13.0 |
| Saratov oblast | 25.0 | 13.0 |
| The Russian Federation | 20.4 | 12.9 |
| Vladimir oblast | 20.7 | 12.9 |
| Orenburg oblast | 30.7 | 12.9 |
| Krasnoyarsk kray | 16.5 | 12.8 |
| Voronezh oblast | 17.7 | 12.8 |
| Lipetzk oblast | 28.2 | 12.8 |
| Chuvash Republic | 28.8 | 12.8 |
| Kaliningrad oblast | 17.5 | 12.7 |
| Samara oblast | 28.7 | 12.7 |
| Moscow city | 18.0 | 12.7 |
| Penza oblast | 27.6 | 12.7 |
| Primorsky kray | 16.1 | 12.6 |
| Khabarovsk kray | 21.8 | 12.6 |
| Ivanovo oblast | 13.1 | 12.5 |
| Sverdlovsk oblast | 20.5 | 12.5 |
| Yaroslavl oblast | 19.9 | 12.5 |
| Kostroma oblast | 18.5 | 12.4 |
| Moscow oblast | 23.4 | 12.4 |
| Novosibirsk oblast | 19.0 | 12.4 |
| Ulyanovsk oblast | 23.1 | 12.4 |
| Nizhny Novgorod oblast | 22.6 | 12.3 |
| Omsk oblast | 25.7 | 12.2 |
| Republic of Tatarstan | 29.6 | 12.1 |
| Udmurt Republic | 21.4 | 12.1 |
| Chelyabinsk oblast | 23.8 | 12.0 |
| Kirov oblast | 20.8 | 11.9 |
| **The Russian Federation** | **20.4** | **12.9** |
